# Three new species of *Mesobiotus* (Eutardigrada: Macrobiotidae) from Sweden with an updated phylogeny of the genus

**DOI:** 10.1038/s41598-025-88063-8

**Published:** 2025-02-06

**Authors:** Sarah Atherton, Jens Hulterström, Roberto Guidetti, K. Ingemar Jönsson

**Affiliations:** 1 Department of Zoology, Naturhistoriska riksmuseet, Box 50007, Stockholm, 104 05 Sweden; 2 Department of Life Sciences, University of Modena and Reggio Emilia, Modena, 41124 Italy; 3 Department of Environmental Science, Kristianstad University, Kristianstad, SE-291 88 Sweden

**Keywords:** Diversity, Taxonomy, Phylogeny, Tardigrades, Macrobiotidae, New species, Biodiversity, Phylogenetics, Speciation, Taxonomy, DNA sequencing

## Abstract

Three new species of *Mesobiotus* (Tardigrada: Eutardigrada: Macrobiotidae) are described from Skåne County in the southernmost region of Sweden. All three species are distinguished morphologically and through differences in DNA sequences as supported by PTP and mPTP analyses. With the addition of *Mesobiotus bockebodicus* sp. nov., *M. skanensis* sp. nov., and *M. zelmae* sp. nov., the number of nominal species of Macrobiotidae in Sweden has increased to 26, 73% of which have been documented from Skåne. Finally, new morphological details and DNA sequences are presented for *Mesobiotus emiliae*, a new record is presented of *M. mandalori* from Sweden, and the phylogenetic relationships within the genus is reconstructed using previously published and new 18S and COI gene sequences.

**Supplementary Information:**

The online version contains supplementary material available at 10.1038/s41598-025-88063-8.

## Introduction

Tardigrada (water bears) is a phylum consisting of small (typically < 1 mm), eight-legged, segmented metazoans that occur in aquatic and limno-terrestrial environments throughout the world. Tardigrades have gained some popularity in recent years due to their charismatic appearances and a unique adaptation called cryptobiosis that enables them to survive under extreme conditions (e.g.^1–3^), and recent studies on their biodiversity (e.g.^4–8^) have increased our knowledge of the phylum significantly. Currently, around 1400 species have been described^9^, although estimates of true tardigrade diversity suggest that many more are yet undiscovered^10^.

The eutardigrade family Macrobiotidae Thulin, 1928^11^ currently comprises up to fifteen genera, several of which were established for different species groups once attributed to its type genus, *Macrobiotus *Schultze, 1834^12^. One of the largest of these genera is *Mesobiotus *Vecchi, Cesari, Bertolani, Jönsson, Rebecchi & Guidetti, 2016^13^, which was erected based on a combination of molecular and morphological data to accommodate species of the *harmsworthi* and *furciger* morpho-groups and currently includes 79 nominal species including four *nomina inquirenda*^14,15^. Animals of *Mesobiotus* are united in having a cuticle without pores, a mouth opening with 10 peribuccal lamellae, a rigid buccal tube with three macroplacoids and a closely positioned (less than its length) microplacoid, Y-type double claws with a common tract with an internal septum, and eggs with conical or hemispherical processes. The exact characteristics separating taxa of the *harmsworthi* group from taxa of the *furciger *group have historically been undefined but were recently clarified by Stec^16^ who proposed adding a third morpho-group (the *montanus* group) and differentiated the three groups based on specific properties of the eggs. Importantly, though the monophyly of *Mesobiotus*as a whole has been confirmed by every phylogenetic investigation since its erection^8,13,17,18^the morpho-groups lack such systematic support and should thus be utilized only informally^16^or abandoned all-together^19,20^.

As with most Macrobiotidae, identifying species of *Mesobiotus *based on morphology alone is often difficult and uncertain despite the existence of an excellent diagnostic key to the species of the genus^14^that is continuously updated with the introduction of new species^16,21^. One of the largest challenges is that the adults are morphologically similar — in some cases identical or distinguishable only by very minute differences in the claws, placoids or oral cavity armature (OCA) — and therefore knowledge of the egg morphology is required for correct identification. Unfortunately, the effort it takes to attain and link an egg to the appropriate adult is not always possible, and the literature is rife with records of uncertain or unidentified specimens qualified with “cf.”, “sp.” or “group sp.” designations (e.g.^4,6,19,22,23^). This is not ideal since large numbers of unrecognized animals or candidate species leads to confusion and hinders discovered taxa from being included in future research or conservation efforts (e.g.^24–28^).

Fortunately, taxonomists have increasingly been describing new tardigrade taxa using an integrative approach that combines molecular tools (phylogeny and genetic delineation) with classical methods (morphology and morphometry) of inference. The utility of DNA sequence data to help identify and delimit species of tardigrades has been well documented (e.g.^29–32^) and is perhaps even more important for species of *Mesobiotus*, since the identity of a single adult or egg specimen could afterwards be ascertained with confidence without the need for cultures or further sampling. Nonetheless, less than a third of the nominal species of *Mesobiotus* currently have DNA sequence data for even a single gene region publicly available.

There are currently 116 species of Tardigrada and 22 species of Macrobiotidae, documented from Sweden, with 50% and 68%, respectively, of those occurring in a single county (Skåne) in the southernmost region of the country. Earlier reports on all Swedish tardigrades were reviewed by Guidetti et al.^33^, and a faunistic study was recently performed that explored the Kristianstads Vattenrike Biosphere Reserve (KVBR), a UNESCO designated area of about 1050 km^2^ located in Skåne. Massa et al.^22^ collected samples from five locations in the KVBR and found a total of 33 species of tardigrades including 12 morphospecies of Macrobiotidae. With the limited number of samples collected but high number of species found (33 species in 34 samples with an average of 3.6 species per sample), they concluded that KVBR is a hotspot for tardigrade diversity and very likely contains many additional unknown species.

The Swedish Species Information Center (ArtDatabanken) commissioned a project in 2001 with the ultimate goal of cataloging all Swedish eukaryotic organisms. As part of this project, we collected several species of Macrobiotidae from locations throughout Skåne and the KVBR. Herein, *Mesobiotus bockebodicus* sp. nov., *Mesobiotus skanensis* sp. nov., and *Mesobiotus zelmae* sp. nov. are described using integrative taxonomy, and DNA sequences from *Mesobiotus emiliae *Massa, Guidetti, Cesari, Rebecchi & Jönsson 2021^22^ as well as a new record of *Mesobiotus mandalori *Erdmann, Kosicki, Kayastha, Mioduchowska & Kaczmarek, 2024^34^ from Sweden are reported for the first time. An updated multilocus phylogeny of the genus is presented.

## Results

### Molecular analyses

**Fig. 1:**
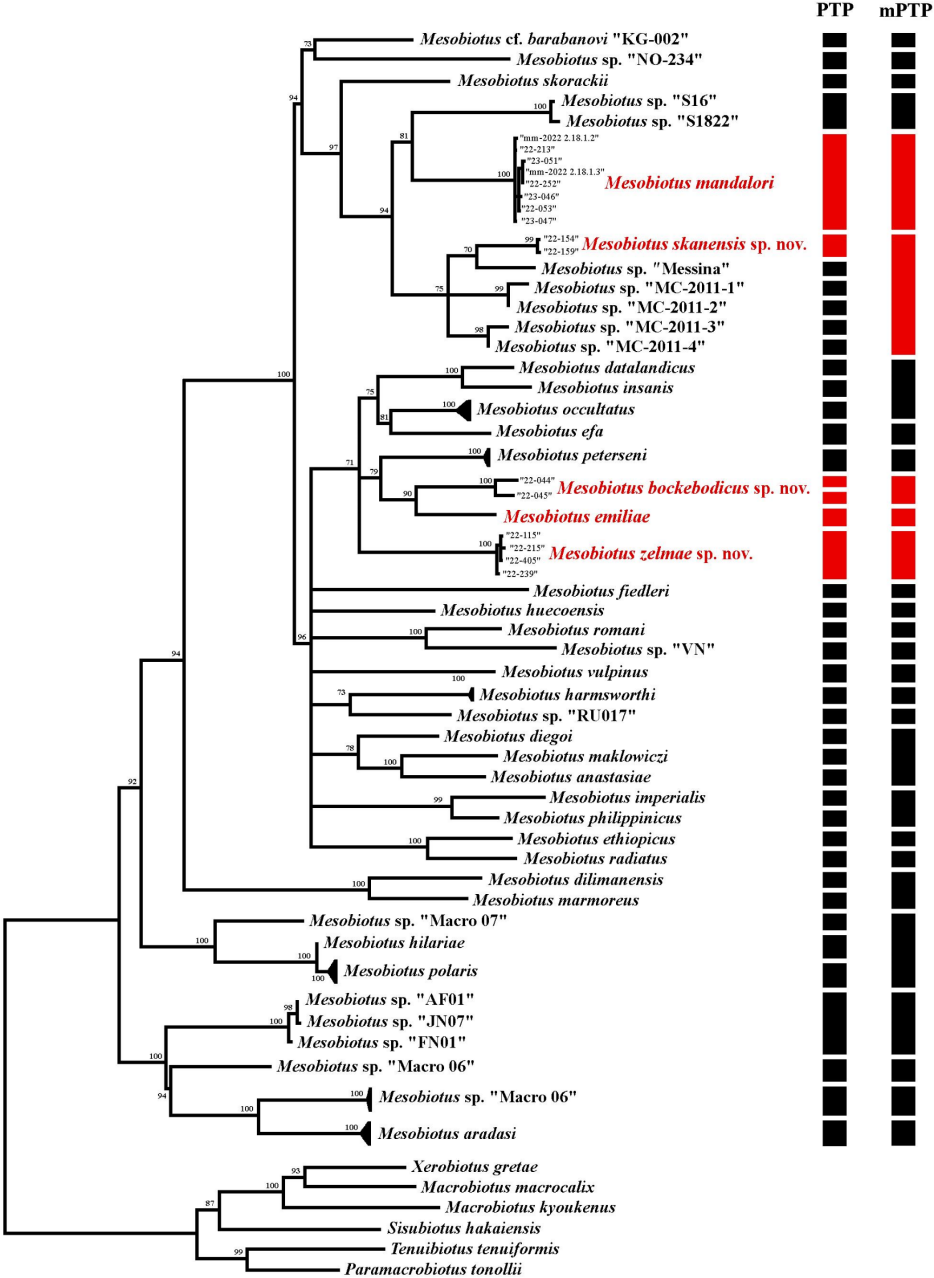
Concatenated 18S and COI gene tree summary with results from the PTP and mPTP analyses. Ultrafast bootstrap support is given at the nodes and species with newly generated genetic data are highlighted in red.

Results from the phylogenetic analyses are presented in Fig. 1and Supplementary Figs. S1–S3. Tree topologies varied only slightly between individual genes and concatenated datasets regardless of whether or not datasets were filtered with GBlocks, with the majority of the differences occurring in the deeper nodes. Many of the deeper nodes were not well supported in the COI gene trees, as could be expected given its relatively fast evolutionary rate^35^.

Figure 1summarizes the COI and 18S concatenated phylogeny. Results were consistent with previous findings^15,16,19^ showing high support (> 0.94) for three main clades of *Mesobiosus*: two smaller clades (those including *Mesobiotus hilariae *Vecchi, Cesari, Bertolani, Jönsson, Rebecchi & Guidetti, 2016^13^ and *Mesobiotus aradasi *(Binda, Pilato & Lisi, 2005)^36^) entirely comprising specimens from Antarctica and one larger clade grouping all non-Antarctic specimens, including the new and newly sequenced species from Sweden. As expected, none of the morpho-groups previously proposed for the genus were recovered as monophyletic, with representatives of each intermixed within all three clades. The non-Antarctic clade was further separated into three well-supported subclades:

1. *Mesobiotus dilimanensis *Itang, Stec, Mapalo, Mirano-Bascos & Michalczyk, 2020^37^ and *Mesobiotus marmoreus *Stec, 2021^38^ united to form a sister group to all the other non-Antarctic specimens;
2. *M.* cf. *barabanovi*, *Mesobiotus skorackii *Kaczmarek, Zawierucha, Buda, Stec, Gawlak, Michalczyk & Roszkowska, 2018^39^, *M. skanensis* sp. nov., *M. mandalori* and eight other undetermined specimens of *Mesobiotus* from Norway (No-234), Finland (S16, S1822) and Italy (MC-2011-1–4, Messina) formed a subclade that was sister to;
3. a generally unresolved subclade that included *M. bockebodicus* sp. nov., *M. emiliae*, *M. zelmae* sp. nov. and seventeen additional species as well as two undescribed specimens of *Mesobiotus* (VN and RU017 from Vietnam and Russia, respectively).

Full results from the mPTP and PTP analyses can be found in Fig. 1. Both analyses supported the delimitation of all three new species, *M. emiliae*, and *M. mandalori* (including specimens from Poland and Sweden). However, the mPTP analysis supported the clade of *M. skanensis* sp. nov. and the five undetermined specimens of *Mesobiotus* from Italy (MC-2011-1–4, Messina) as a single species, while the PTP analysis separated the clade into six different species (*M. skanensis* sp. nov., MC-2011-1, MC-2011-2, MC-2011-3, MC-2011-4, and Messina). Detailed morphological data was not provided for the unidentified specimens of *Mesobiotus* from Italy (note that they were originally identified as members of the “*Macrobiotus harmsworthi* group” *sensu *Ramazzotti & Maucci^40^, and thus any more modern definitions of the morpho-group—and the morphological data that would subsequently be implied—cannot necessarily be attributed to these animals), but the large differences and distances between the collection locations support treating the Swedish specimens separately from the Italian specimens at this time.

Similarly, while the mPTP analysis united all specimens of *M. bockebodicus* sp. nov. as a single species, PTP analysis separated the four specimens in two potential species: specimens 22–044, 22–048 and 23–030 as one species, specimen 22–045 as a second species. Specimen 22–045 had a COI haplotype that differed from the haplotype of the other three specimens by 6.0%, which is higher than the COI intraspecific distances of the other species of the genus (0.0 – 4.5%; Supplementary Table S1) but lower than the COI interspecific distances within the genus (15.0 – 28.3%; Supplementary Table S1). However, several other factors do not support specimen 22–045 as a separate species: the 18S and 28S sequences for all four specimens were identical; specimen 22–045 was collected from the same sample as the other specimens, and no morphological or morphometric differences were identified that distinguished specimen 22–045 from the other specimens of *M. bockebodicus* sp. nov. For these reasons, specimen 22–045 is represented as a more distant haplotype of *M. bockebodicus* sp. nov. rather than a separate, closely related species.

## Taxonomy

Family Macrobiotidae Thulin, 1928.

Genus *Mesobiotus*Vecchi, Cesari, Bertolani, Jönsson, Rebecchi & Guidetti, 2016.

***Mesobiotus emiliae*Massa**,** Guidetti**,** Cesari**,** Rebecchi & Jönsson**,** 2021**.

Figures 2 and 3.

**Fig. 2:**
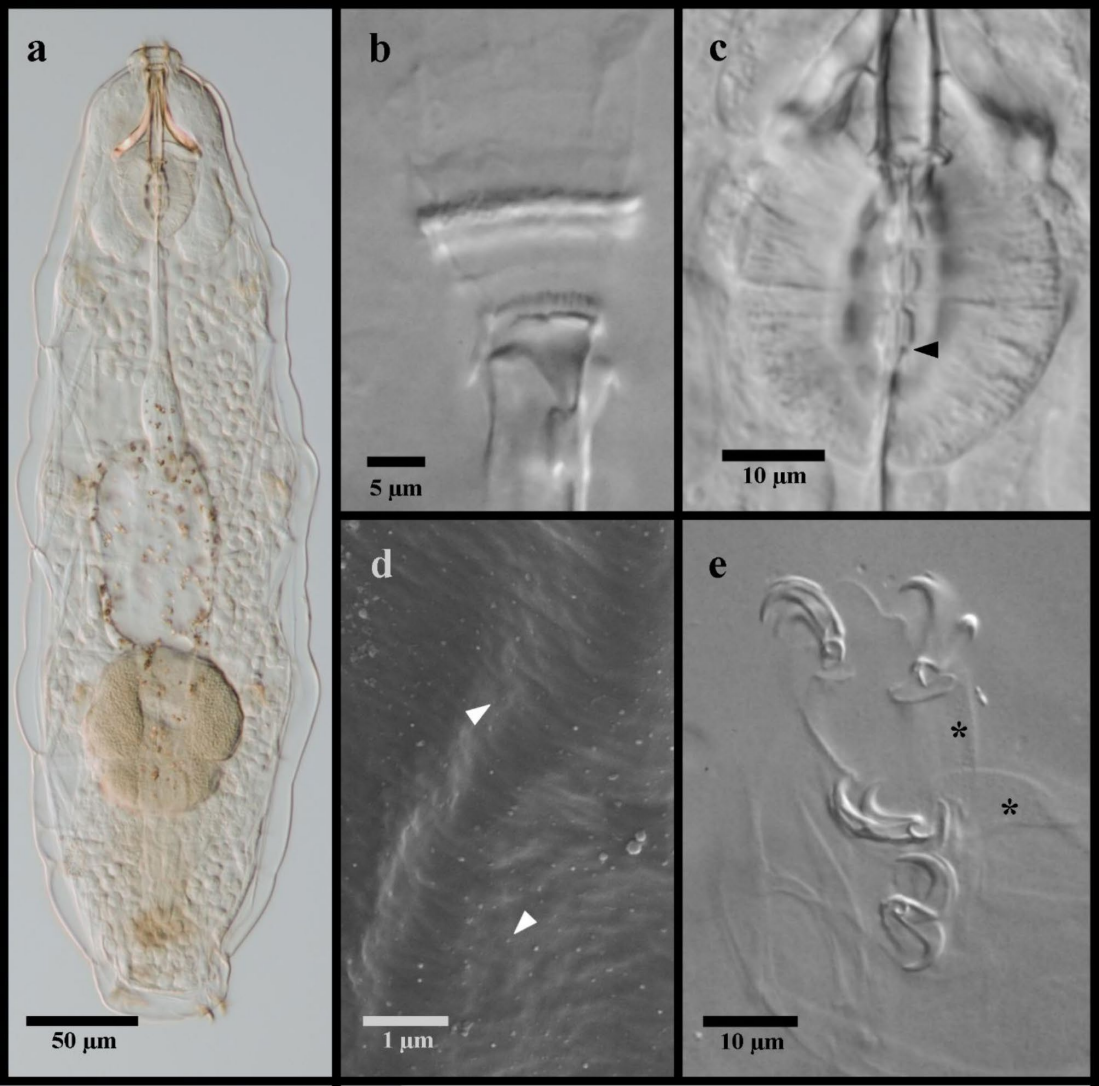
Mesobiotus emiliae. (**a**) whole body, live animal in water; (**b**) ventral oral cavity armature in Hoyer’s medium; (**c**) placoid morphology, animal in water; (**d**) SEM image of the body cuticle; (**e**) claws and legs IV in Hoyer’s medium, asterisk indicates the larger granulation visible in LM. Black flat arrowhead indicates the constriction of the third macroplacoid; white flat arrowhead indicates examples of the fine granulation present over the entire cuticle.

*Materials examined*. A total of five animals and five eggs were examined and documented with digital video and micropictographs, including two animals and one egg mounted on microscope slides in Hoyer’s medium, and three animals and three eggs processed for DNA sequencing. In addition, twenty paratypes (nineteen adults and an egg) in Hoyer’s medium from the collection of the Swedish Natural History Museum in Stockholm, Sweden were examined, and a specimen from the previous study of Massa et al.^22^ observed with SEM.

**Fig. 3:**
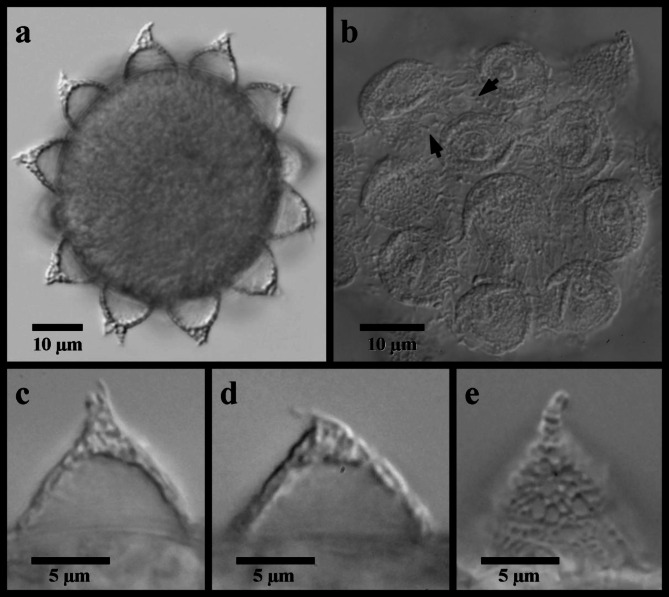
*Mesobiotus emiliae* eggs. (**a**) whole egg at the midsection; (**b**) egg processes and surface; (**c**,**d**,**e**) examples of egg processes; black full arrow indicates crown of thickening at the base of the processes.

*Type Locality*. Sweden, Skåne, Sånnarna, 55°55’44.2’’N 14°14’46.7’’E, moss on sandy soil, collected on 22 March 2022.

*Additional Localities*.

-  Sweden, Skåne, Fjälkestad, 56°08’58.3’’N 14°11’46.5’’E, moss on a rock, collected 23 March 2022;
-  Sweden, Skåne, Bockeboda recreational area, 56°01’38.7’’N 13°59’33.9’’E, moss on a rock, collected on 22 March 2022;
-  Sweden, Skåne, Simrishamn, 55°38’43.8’’N 14°11’30.1’’E, moss on a rock, collected 20 May 2022.

*Description*. Body mostly transparent without eyespots in living animals, 290–380 μm long (Fig. 2a). Body cuticle smooth under LM with minute granules present over the entire cuticle visible only with SEM (Fig. 2d). Leg granulation composed by larger granules only visible on the fourth pair of legs with LM (Fig. 2e). OCA of *harmsworthi *type^14^ without supplementary teeth (Fig. 2b). Pharyngeal bulb with rows of three macroplacoids and closely situated microplacoid (Fig. 2c). Macroplacoid length sequence 1 < 2 < 3. Claws increasing in size posteriorly, with those of the fourth pair of legs clearly larger than the others (Fig. 2e). Smooth lunules present under all claws. Eggs 62.4–72.0 μm full diameter, with 11–12 processes on the circumference (Fig. 3a). Processes shaped as wide cones (base: height 1.42–1.64) with short, flexible tips; reticulated (Fig. 3a–e). Process bases with crown of ridges (Fig. 3b). Egg surface between wrinkled or sparsely dotted.

*DNA Sequences*. Sequences for *M. emiliae* were attained for all three molecular markers and from three adult animals and two eggs. All markers were represented by a single haplotype, with:

- 18S: 1769 bp, GenBank accession number PQ367886;
- 28S: 921 bp, GenBank accession number PQ367892;
- COI: 658 bp, GenBank accession number PQ365768.

*Remarks*. One adult specimen deviated from what was previously reported in a larger body size (380 μm vs. max. 342 μm) and slightly longer anterior claws on the fourth legs (*pt* 27.6 vs. max. *pt* 26.0), but the larger specimen as well as all other adult and egg specimens corresponded in all other ways to the original description of *M. emiliae*. All specimens were collected from the type location. DNA sequences of three molecular markers are newly provided for this species, as DNA amplicons could not originally be attained from the type materials. The observation with SEM of the specimen prepared by Massa et al.^22^ has showed very minute granules regularly distributed over the entire body cuticle (Fig. 2d).

***Mesobiotus mandalori *Erdmann**,** Kosicki**,** Kayastha**,** Mioduchowska & Kaczmarek**,** 2024**.

Figures 4, 5 and 6.

*Materials examined*. A total of 22 animals and five eggs were analyzed and documented with digital video and micropictographs, including: eight animals and three eggs mounted on microscope slides in Hoyer’s medium; two animals fixed on SEM stubs; and twelve animals and two eggs processed for DNA sequencing.

**Fig. 4:**
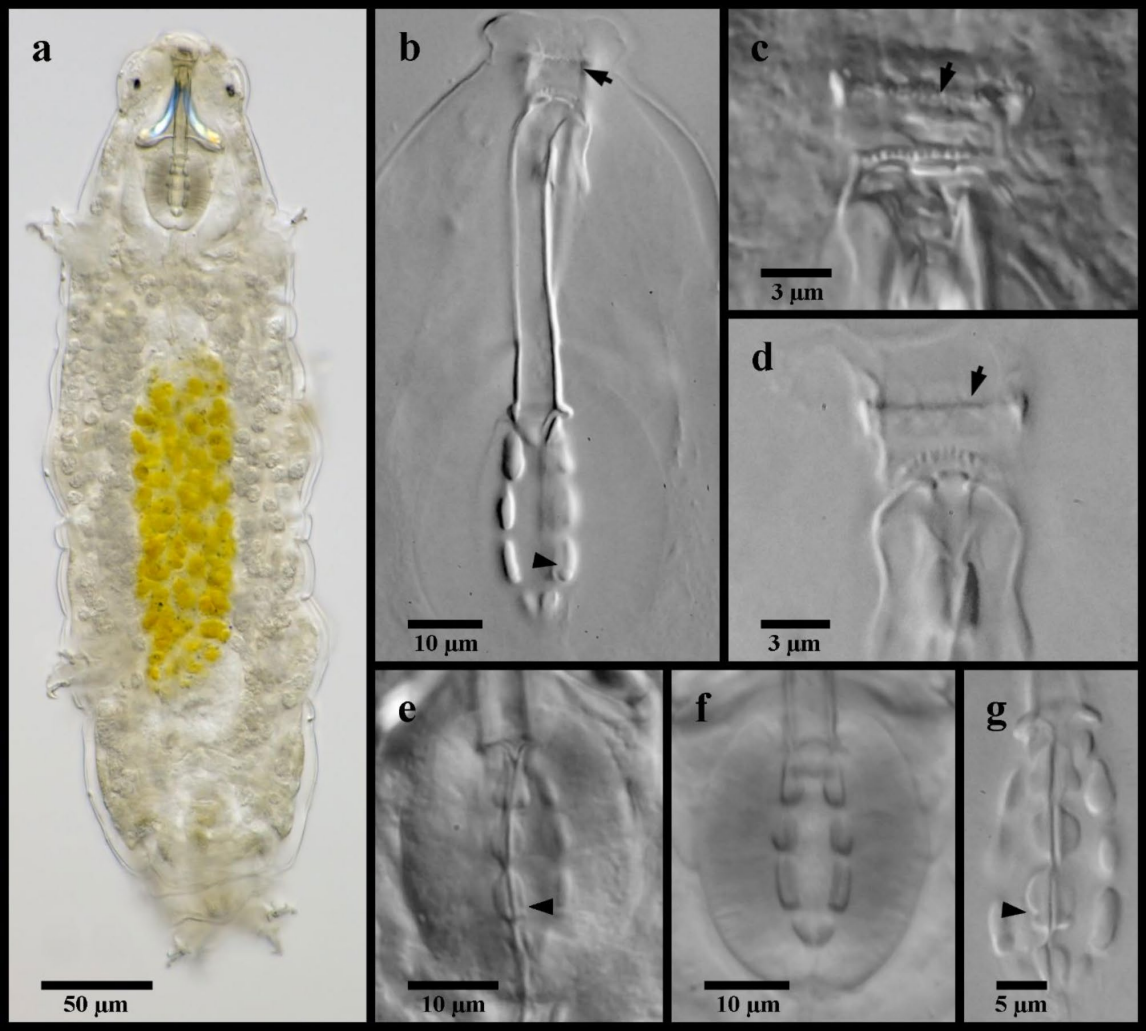
*Mesobiotus mandalori* from Sweden (**a**) whole body, live animal in water; (**b**) bucco-pharyngeal apparatus, lateral view in Hoyer’s medium; (**c**) dorsal oral cavity armature in water; (**d**) ventral oral cavity armature in Hoyer’s medium; (**e**) placoid morphology, dorsal view in water; (**f**) placoid morphology, lateral view in water; (**g**) placoid morphology, dorsal view in Hoyer’s medium. Black full arrows indicate first band of teeth; black flat arrowheads indicate the location of the constriction of the third macroplacoid.

**Fig. 5:**
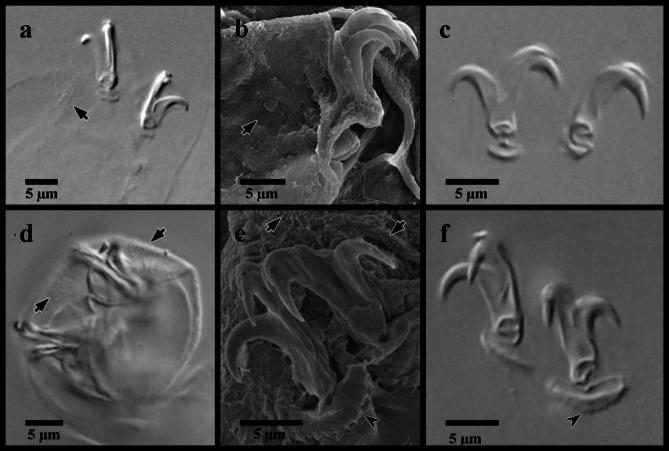
*Mesobiotus mandalori* from Sweden claws. (**a**) claws of leg II in Hoyer’s medium; (**b**) SEM image of claws and leg II; (**c**) claws of leg I in Hoyer’s medium; (**d**) legs IV in water; (**e**) SEM image of the claws of leg IV; (**f**) claws of leg IV in Hoyer’s medium. Black full arrows indicate granulation present on all legs; black indented arrowheads indicate the dentate lunules of the claws of leg IV.

**Fig. 6:**
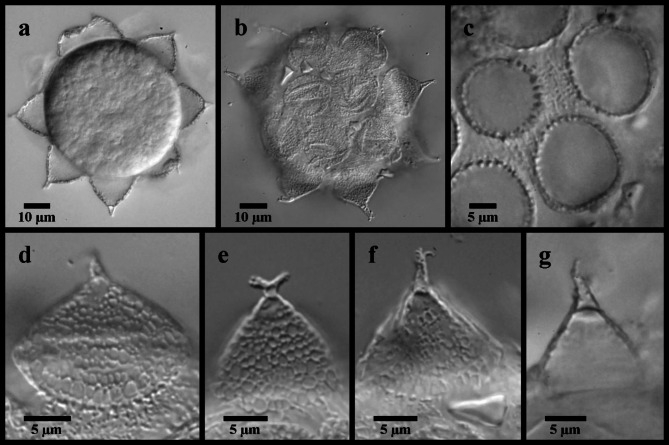
*Mesobiotus mandalori* from Sweden eggs. (**a**) whole egg at the midsection; (**b**) surface of the egg and egg processes; (**c**) bases of the processes and egg interprocess surface; (**d**,**e**,**f**,**g**) examples of egg processes.

*Localities*.

Sweden, Skåne, Bockeboda recreational area, 56°01’53.9’’N 13°59’30.4’’E, leaf litter primarily from *Corylus avellana*L. 1753 (common hazel), collected on 21 March 2022;

- Sweden, Skåne, Fjälkestad, 56°08’58.3’’N 14°11’46.5’’E, one sample of needle litter from *Picea abies* (L.) Karst 1881 (spruce) and one sample of moss from the bark of a spruce tree taken from ~ 1.5 m high, collected on 23 March 2022;
- Sweden, Skåne, Forestad, 56°01’25.2’’N 13°20’51.9’’E, moss on a stone wall, collected on 17 April 2022;
- Sweden, Skåne, Simrishamn, 55°38’43.8’’N 14°11’30.1’’E, leaf litter primarily from *Fagus sylvatica* L., 1753 (European beech), collected on 20 May 2022;

*Description of Swedish population*. Body transparent, 285–465 μm long (Fig. 4a). Black eye-spots present in living animals and in animals fixed in Hoyer’s medium. Body cuticle smooth with evident granulation on legs I–IV (Fig. 5a, b, d). OCA of *harmsworthi* type without accessory teeth (Fig. 4b–d). Pharyngeal bulb with three macroplacoids and closely situated microplacoid (Fig. 4b, e–g). Macroplacoid length sequence 2 < 3 < 1. Lunules under claws on legs I–III smooth (Fig. 5c), on legs IV dentate (Fig. 5e–f). Eggs 78.1–83.8 μm full diameter, with 10 processes on the circumference (Fig. 6a). Each egg process shaped as a wide cone (base: height 1.08–1.19) with a short terminal tip and up to a few very short filaments, appearing reticulated with bubbles in LM (Fig. 6b, d–g). Process base without areolation (Fig. 6b–c). Egg surface appears granulated in LM (Fig. 6c).

*DNA Sequences*. Sequences from the Swedish populations of *M. mandalori* were attained for all three molecular markers from twelve adult animals. 28S and 18S were represented by a single haplotype, and COI was represented by six haplotypes (range uncorrected p-values between COI haplotypes 0.15–0.76%):

- 18S: 1769 bp, GenBank accession number PQ367887;
- 28S: 921 bp, GenBank accession number PQ367893;
- COI haplotype-1: specimens 22–053 and 23–049; 658 bp; GenBank accession number PQ365769;
- COI haplotype-2: specimen 22–213; 658 bp; GenBank accession number PQ365770;
- COI haplotype-3: specimens 22–252, 22–055; 658 bp; GenBank accession number PQ365771;
- COI haplotype-4: specimens 23–046 and 23–048; 658 bp, GenBank accession number PQ365772;
- COI haplotype-5: specimens 23–047, 22–060 and 22–054; 658 bp; GenBank accession number PQ365773;
- COI haplotype-6: specimens 23–051 and 23–052; 658 bp; GenBank accession number PQ365774.

*Remarks*. Though the Swedish populations generally matched the original description of *M. mandalori *from central Poland^34^, small variations were present in three specimens: one specimen collected from Bockeboda had a larger body size (455 μm vs. max 447 μm) and larger claws (*pt* for external primary claw I/II/IV 26.8/30.2/33.6 vs. max. 25.4/27.7/28.3); one specimen from Fjälkestad had a larger body size (465 μm) and larger third macroplacoids (*pt* 16.5 vs. max. 16.1); and one specimen from Bockeboda had a longer stylet insertion point (*pt* 79.8 vs. max. 78.6) and wider buccal tube (*pt* 13.4 vs. max. 11.5). These variations are attributed to normal intraspecific variation, and DNA sequences were recovered from each of these specimens and their identities supported with PTP and mPTP analyses. The ranges of the uncorrected p-distances between the Swedish populations of *M. mandalori* and the Polish type population is 0.00–0.49% for COI, while the 18S sequences were identical for all specimens regardless of sampling location.

***Mesobiotus bockebodicus*** **sp. nov.**

Figures 7 and 8.

**Fig. 7:**
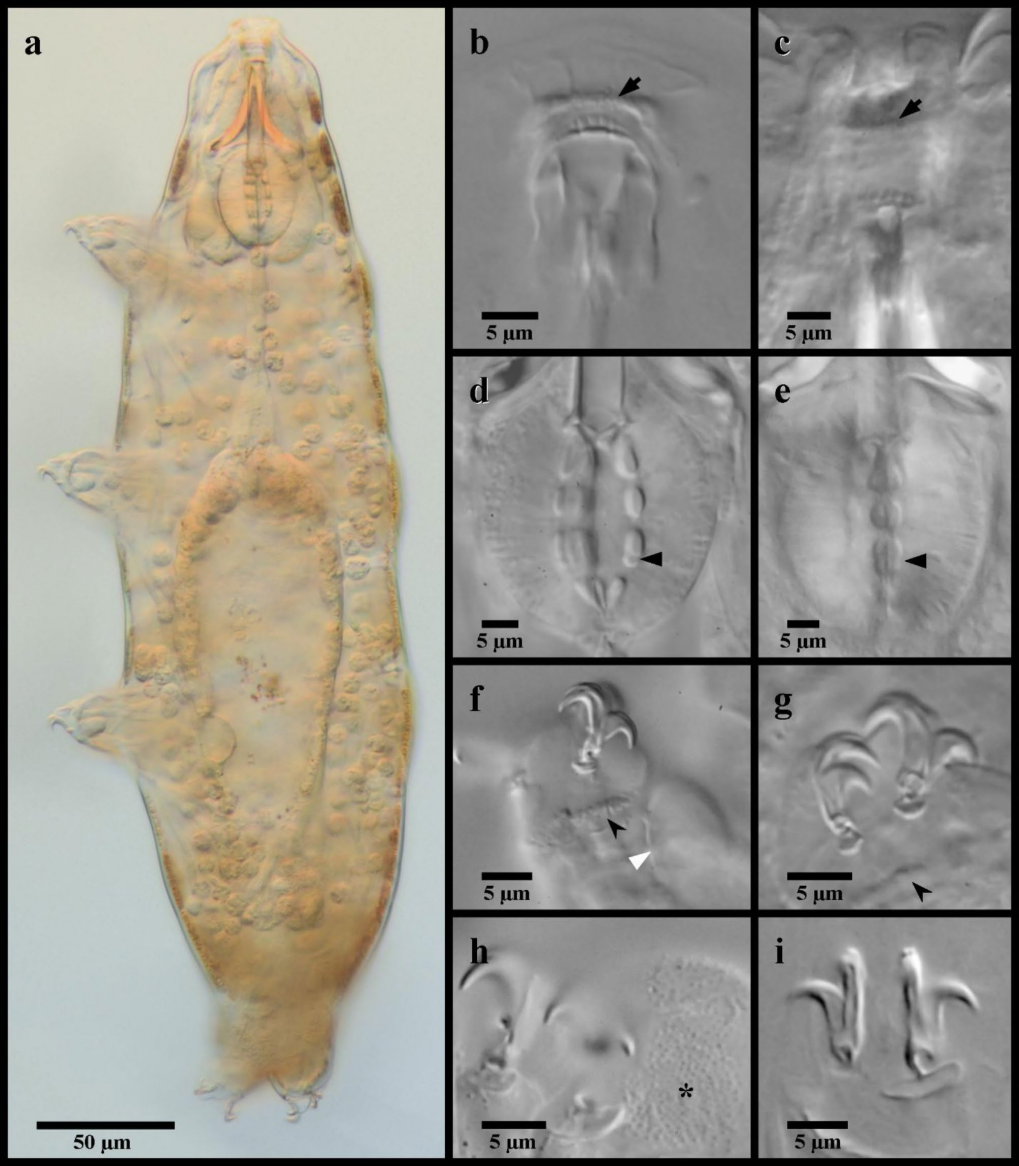
*Mesobiotus bockebodicus* sp. nov. (**a**,**d**,**f**) photo of live holotype specimen 22–046 in water; (**b**,**i**) holotype specimen 22–046 in Hoyer’s medium; (**c**,**e**) specimen 22-047f in water; (**g**,**h**) specimen 24–076 in water. (**a**) whole body; (**b**) dorsal oral cavity armature; (**c**) ventral oral cavity armature; (**d**) placoid morphology, lateral view; (**e**) placoid morphology, dorsal view; (**f**) claws and leg I; (**g**) claws and leg III; (**h**,**i**) claws and legs IV. Black full arrows indicate first band of teeth; black flat arrowheads indicate the location of the constriction of the third macroplacoid; black indented arrowheads indicate the cuticular bars; the white flat arrowhead indicates the cuticular bulge; the asterisk indicates the large granulation present on legs IV.

**Fig. 8:**
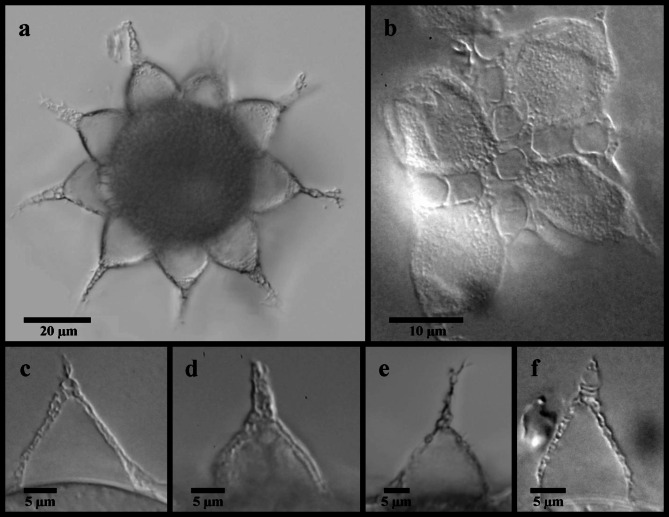
*Mesobiotus bockebodicus* sp. nov. eggs. (**a**,**d**,**e**) specimen 22–048; (**b**,**f**) specimen 22–039; (**c**) specimen 22–043; (**a**) whole egg at the midsection; (**b**) egg surface; (**c**,**d**,**e**,**f**) examples of egg processes.

*Zoobank registration*. urn:lsid:zoobank.org:act:4CAC341E-9F49-456D-A21B-3277E6A69C5F.

*Materials examined*. A total of 16 animals and nine eggs were analyzed and documented with digital video and micropictographs, including five animals and four eggs mounted on microscope slides in Hoyer’s medium and eight animals and four eggs processed for DNA sequencing.

*Type Depositories*. The holotype (specimen 22–046; slide SMNH-Type-1555) and two paratypes (specimens 24-003f and 24-005f; slides SMNH-Type-1556–7), and three eggs (specimens 22 − 014, 22–043-2 and 22–050-1; slides SMNH-Type-1558–9) are deposited at the Swedish Natural History Museum in Stockholm, Sweden. Two paratypes (specimens 22 − 013 and 24-002f) and one egg (specimen 22–039) are in the Bertolani Collection of University of Modena and Reggio Emilia (Italy).

*Type locality*. Sweden, Skåne, Bockeboda recreational area. 56°01’38.7’’N 13°59’33.9’’E, leaf litter primarily from European beech, collected on 22 March 2022 (Supplementary Fig. S4).

*Additional locality*. Sweden, Skåne, Bockeboda recreational area, 56°01’38.7’’N 13°59’33.9’’E, moss from a rock, collected on 22 March 2022.

*Diagnosis*. Species of *Mesobiotus*, light brown in LM without eye-spots, 225–400 μm long. Cuticle smooth with evident granulation on legs IV. OCA of *harmsworthi* type without accessory teeth. Pharyngeal bulb with rows of three macroplacoids and closely situated microplacoid. Macroplacoid length sequence 2 < 1 < 3. Claws and lunules on legs I-III of similar size, larger on leg IV. Lunules smooth. Eggs 81.6–99.6 μm full diameter, with 7–9 processes on the circumference. Processes in the shape of tall cones (base: height 0.62–0.89) with long slender endings and few short filaments, reticulated with bubbles. Full areolation, with approximately nine areolae surrounding each process (alternating one or two areolae between neighboring processes). Egg surface within areolae mostly smooth.

*Etymology*. The species is named after the Bockeboda recreational area, within which the animals and eggs were collected.

*Description*. Living animals light brownish without eyespots (Fig. 7a), transparent to mostly opaque in LM and fully transparent without or with much less color after fixation in Hoyer’s medium. Body length 225–400 μm. Cuticle smooth without sculpturing, gibbosities, spines, papillae or pores, but with granulation on leg pair IV visible in LM (Fig. 7h). Granulation on leg pairs I-III absent. A cuticular bulge (pulvinus) is present on the internal surface of leg pairs I-III (Fig. 7f).

Bucco-pharyngeal apparatus of the *Macrobiotus *type^41^, with terminal mouth and ten peribuccal lamellae.

OCA of *harmsworthi* type without supplementary teeth (Fig. 7b–c), comprising: (1) an anterior band of small granules at the base of the peribuccal lamellae, (2) a single posterior row of teeth in the shape of vertical (= parallel to the buccal tube) ridges located just anterior to the third band of teeth and (3) three dorsal and three ventral transversal crests just before the buccal tube opening. Transversal crests on the dorsal side shaped as bars, with the paired lateral crests wider than the medial crest. Ventral crests thicker, with the medial crest rounded and two lateral crests more triangular.

Buccal tube rigid with ventral lamina 16.7–27.0 μm (*pt* 60.6–67.0) long. Stylet supports inserted 19.3–36.2 μm (*pt *73.7–78.7) from the anterior end of the buccal tube, leading to typically-shaped stylet system^42^. Posterior end of the buccal tube within the pharyngeal bulb, terminating in a distinct thickening. Pharynx with triangular apophyses and rows of three macroplacoids and a single microplacoid (Fig. 7d–e). In frontal view, first macroplacoid drop shaped; second rounded; and third paddle-shaped with an evident constriction ~ 2/3 of the way from the anterior leading to a rounded posterior end. Length of first, second and third macroplacoids 4.6–7.5 μm (*pt* 12.7–16.9), 3.7–5.0 μm (*pt* 10.3–12.6), and 4.9–8.6 μm (*pt* 13.7–19.7), respectively. Length sequence 2 < 1 < 3. Microplacoids triangular, 3.0–4.0 μm (*pt* 7.4–10.1) long and situated closely posterior to the third macroplacoids.

Double-claws of the *Mesobiotus *type^13^, with evident accessory points on the primary branch. Claws of the first, second and third legs (Fig. 7f–g) similar in sizes (Table 1), the external slightly larger than the internal. Claws of the fourth legs (Fig. 7h–i) larger with larger lunules than those of the other legs, with the posterior claws/lunules even larger than the anterior claws/lunules. Lunules of all claws smooth. A single, continuous cuticular bar present on each of the first three pairs of legs below the lunules (Fig. 7f–g).

**Table 1:** Morphometric data for selected morphological characters of the new species, including the range of the absolute measurements (µm) and the range of the measurements relative to the buccal tube length (*pt*).

	*M. bockebodicus* sp. nov. (*n* = 16)		*M. skanensis* sp. nov. (*n* = 20)		*M. zelmae* sp. nov. (*n* = 24)	
	µm	pt	µm	pt	µm	pt
Body length	225–400	730–1062	430–570	910–1219	215–395	775–1145
Bucco-pharyngeal apparatus						
-Buccal tube length	26.2–41.8		43.5–53.6		27.5–39.3	
-Stylet support insertion	19.8–32.8	75.5–78.7	34.8–43.0	77.5–80.7	21.5–31.2	74.5–79.5
-Buccal tube external width	5.1–7.0	14.4–19.5	7.0–9.2	15.2–19.6	4.7–6.9	16.1–19.1
-Buccal tube internal width	3.2–4.9	9.0–12.2	4.1–7.0	9.3–15.1	2.3–4.1	8.5–13.2
-Ventral lamina length	16.7–27.0	63.2–67.0	30.0–37.9	65.7–71.1	17.3–25.6	56.9–71.1
Placoid Lengths						
-Macroplacoid 1	4.6–6.7	12.7–16.9	7.1–9.7	14.0–19.5	3.7–5.7	12.5–14.8
-Macroplacoid 2	3.7–5.0	10.3–12.6	5.0–8.2	11.3–15.4	3.1–4.9	10.5–13.0
-Macroplacoid 3	4.9–7.8	13.7–19.7	7.8–9.7	14.6–19.4	3.7–6.1	13.6–15.9
-Microplacoid	3.0–4.1	8.4–10.8	4.1–5.9	8.6–12.4	2.9–4.5	9.9–13.0
-Macroplacoid row	14.1–18.4	39.5–45.7	21.5–26.5	43.7–52.6	11.5–18.0	41.7–47.1
-Placoid row	17.9–23.4	50.1–58.1	25.8–30.8	55.2–61.6	14.6–22.4	53.1–60.0
Claw I lengths						
-External primary	7.6–10.2	19.1–29.0	10.1–12.9	21.2–27.9	5.8–8.6	18.9–24.6
-External secondary	5.6–8.5	13.9–23.3	6.6–11.5	13.2–23.5	4.6–7.6	15.3–20.1
-Internal primary	7.5–10.2	18.6–29.4	9.8–12.8	20.9–26.8	5.5–8.1	18.2–23.5
-Internal secondary	5.7–8.5	14.4–21.8	7.6–10.8	16.8–23.3	4.5–6.8	15.3–19.5
Claw II/III lengths						
-External primary	7.8–10.1	19.6–31.7	11.1–14.8	22.4–32.0	5.7–9.4	18.7–27.2
-External secondary	5.5–7.4	13.6–26.3	8.2–12.2	16.4–23.3	4.5–7.9	14.8–21.3
-Internal primary	7.5–11.1	18.6–29.0	10.4–14.0	21.3–29.4	6.0–8.6	19.7–25.7
-Internal secondary	5.5–8.9	13.9–21.8	7.8–12.4	15.6–26.5	4.9–7.5	16.0–20.5
Claw IV lengths						
-External primary	8.3–11.3	20.6–28.9	11.7–15.6	26.4–33.7	6.1–10.3	22.2–29.9
-External secondary	6.3–9.1	15.6–25.5	8.5–12.9	19.2–27.9	4.6–7.8	16.8–21.7
-Internal primary	9.4–12.1	23.3–30.6	12.6–16.0	28.4–34.6	6.3–10.8	22.9–29.3
-Internal secondary	6.5–9.4	16.1–24.1	9.1–12.3	20.5–26.6	4.9–7.8	18.0–21.2

Single ovary located dorsal-caudal to the intestine, occasionally with one to three well-developed eggs. Testes, seminal vesicles and seminal receptacles not seen.

Globular eggs laid freely, ornamented, 81.6–99.6 μm in diameter including processes (Fig. 8; Table 2). Egg circumference with 7–9 processes (Fig. 8a). Processes in the shape of cones with long slender endings, often terminating with one or more very short filaments (Fig. 8c–f). Process height 18.5–28.7 μm and diameter at the base 13.7–19.7 μm (base: height 0.62–0.89). Process walls with internal and external sides interspersed with trabecular structures that cause reticulated appearance in LM with bubble-like structures in the longer process tips. The bases of neighboring processes fully connect (= full areolation *sensu* Kaczmarek et al.^14^), with any given process forming an alternating pattern of one or two areoles between each of its neighboring processes, for a total of ~ nine areoles around each process (Fig. 8b). Ridges delimiting areolae reticulated, and the egg surface between the ridges (surface of the areolae) mostly smooth, with only sparse wrinkles.

**Fig. 9:**
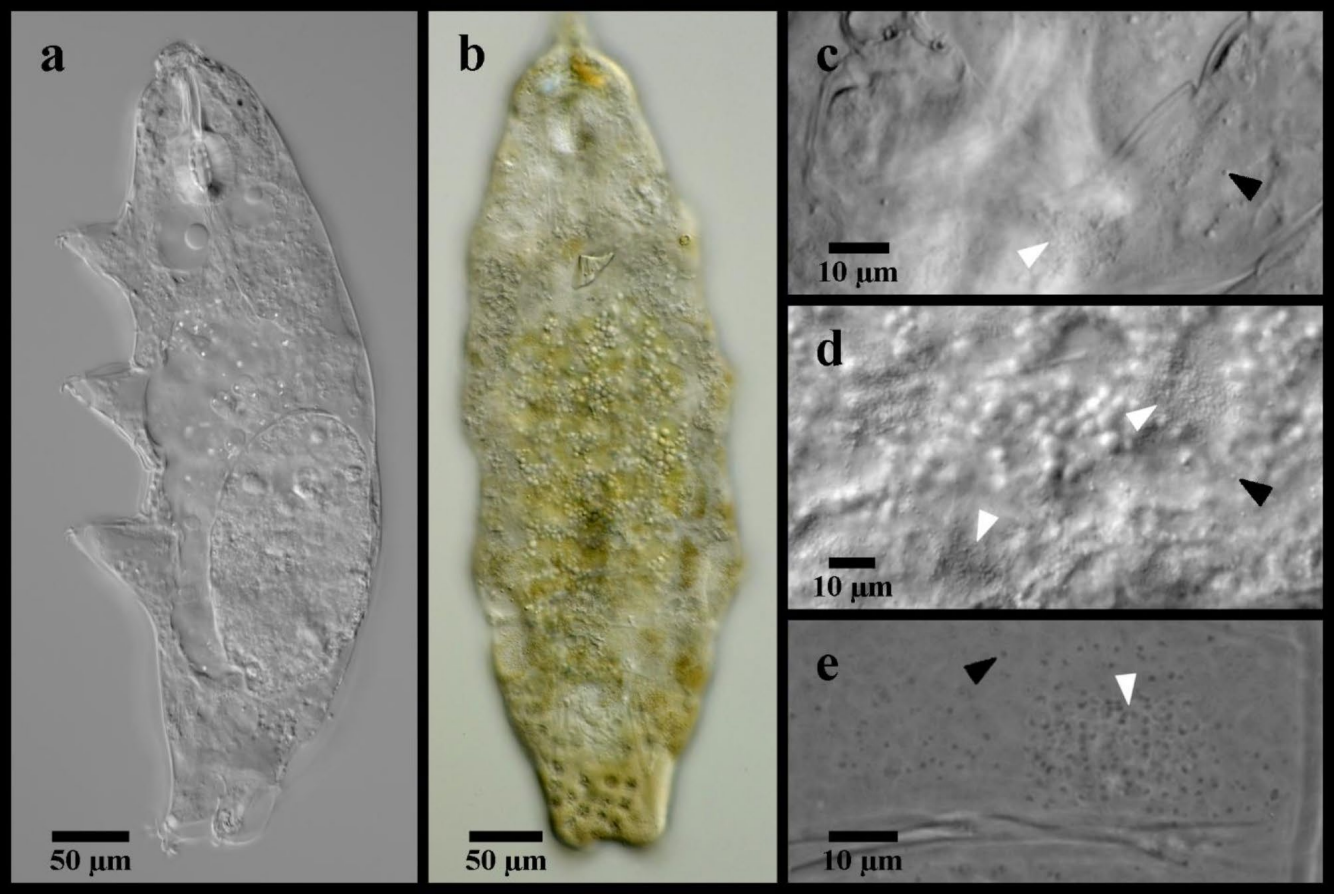
*Mesobiotus skanensis* sp. nov. (**a**,**c**) holotype specimen 22–163-1, DIC photo of live animal in water; (**b**,**d**) specimen 22–159 in water, DIC; (**e**) paratype specimen 22-118af in Hoyer’s medium, phase contrast. (**a**) whole body; (**b**) whole body with a focus on the dorsal body cuticle; (**c**) ventral body cuticle; (**d**,**e**) dorsal body cuticle. The white flat arrowheads indicate examples of the patches of medium-sized granules that appeared present on the body cuticle; the black flat arrowheads indicate examples of individual granules present between the granule patches.

**Table 2:** Morphometric data for the eggs of the new species, including the number of measurements for each character, the range of the measurements in µm and the mean and standard deviation.

	*M. bockebodicus* sp. nov.			*M. skanensis* sp. nov.			*M. zelmae* sp. nov.		
	*N*	Range (µm)	Mean ± SD	*N*	Range (µm)	Mean ± SD	*N*	Range (µm)	Mean ± SD
Egg bare diameter	8	44.9–69.0	54.5 ± 9.3	5	61.0–78.0	70.6 ± 6.2	7	46.1–51.9	49.3 ± 2.2
Egg full diameter	7	81.6–99.6	92.4 ± 7.3	5	91.2–106.6	100.1 ± 6.2	7	71.7–81.3	76.2 ± 3.0
Process height	22	18.5–28.7	22.1 ± 3.3	15	12.8–19.0	15.0 ± 1.8	20	11.8–15.8	14.1 ± 1.1
Process base	24	13.7–19.7	16.6 ± 1.4	15	12.4–17.9	15.5 ± 1.8	20	10.2–14.6	12.3 ± 1.3
Base height	22	0.62–0.89	0.76 ± 0.08	15	0.84–1.36	1.04 ± 0.14	20	0.67–0.96	0.87 ± 0.08
Interprocess distance	24	2.0–5.5	3.7 ± 0.9	15	3.0–5.6	4.0 ± 0.7	12	2.6–3.7	3.1 ± 0.4
#Processes on circumference	8	7–9	8.4 ± 0.7	5	11–12	11.5 ± 0.6	7	10–12	11.1 ± 0.9

*DNA Sequences*. Sequences for *M. bockebodicus* sp. nov. were attained for all three molecular markers and from three adult animals and one egg. 18S and 28S loci were represented by a single haplotype, while COI resulted in two haplotypes (uncorrected p-value between COI haplotypes 6.31%):

- 18S: 1769 bp, GenBank accession number PQ367888;
- 28S: 921 bp, GenBank accession number PQ367894;
- COI haplotype-1: specimens 22–044, 22–048 and 23–030; 658 bp; GenBank accession number PQ365775;
- COI haplotype-2: specimen 22–045; 658 bp; GenBank accession number PQ365776.

*Taxonomic Remarks*. Specimens of *M. bockebodicus* sp. nov. were collected in samples where specimens of *M. emiliae* were also found, and the latter species was also found to be its sister species (Fig. 1). Though the two species are clearly distinguished molecularly and by the egg morphologies, the adults appear very similar and thus require cautious morphological identification. However, animals of *M. bockebodicus* sp. nov. do differ from *M. emiliae* by their brown pigmentation, the generally longer ventral lamina (*pt* 63.2–67.0 in the new species vs. *pt* 54.5–63.7), the longer first macroplacoid (*pt* 12.7–16.9 in the new species vs. *pt* 7.8–12.6), the macroplacoid length sequence (2 < 1 < 3 vs. 1 < 2 < 3), and the shape of the third macroplacoids, which are rounded and not as narrow posterior to the constriction in the new species (e.g. compare Figs. 2c and 7e).

*Mesobiotus bockebodicus* sp. nov. displays *harmsworthi* type OCA without supplementary teeth and possesses eggs with reticulated, conical processes and areolation. This condition is similarly found in seven other species of *Mesobiotus*, but *M. bockebodicus* sp. nov. can be distinguished:

1. from *Mesobiotus barabanovi* (Tumanov, 2005)^43^ by a smaller body length (225–400 μm for the new species vs. 452.2–591.1 μm), the absence of eyespots in living animals, the smaller claws (e.g. claws of leg I and IV, respectively, *pt* 19.1–29.0 and 20.6–30.6 vs. *pt* 32.3–44.1 and 51.7–66.7), the presence of smooth lunules under the claws of leg IV, and the smaller eggs (44.9–69.0 μm vs. 81.2–91.3) with complete areolae (= full areolation vs. semi-areolation), longer processes (18.5–28.7 μm vs. 11.0–17.2 μm) with wider bases (13.7–19.7 μm vs. 8.5–12.7 μm), and fewer processes on the egg circumference (7–9 vs. ~ 20);
2. from *Mesobiotus barbarae* (Kaczmarek, Michalczyk & Degma, 2007)^44^ by the longer lamina (*pt* 63.2–67.0 for the new species vs. *pt* 58.2–63.2), the different shape of the first macroplacoids (with more tapered anterior ends), the absence of granulation on legs II and III, the presence of smooth lunules under the claws of leg IV, the smaller eggs (full diameter 81.6–99.6 μm vs. 106.0–115.0 μm) with fewer processes on the egg circumference (7–9 vs. 10), more numerous areolae around each process (9 vs. 5–6) and mostly smooth egg shell inside the areolae (vs. small-dotted, irregular design);
3. from *Mesobiotus ethiopicus *Stec & Kristensen, 2017^45^ by the presence of longer third than first macroplacoids (macroplacoid length sequence 2 < 1 < 3 for the new species vs. 2 < 3 < 1), the different shapes of the first and third macroplacoids (first macroplacoids with a more tapered anterior end and third macroplacoids shaped slightly convex rather than concave prior to the subterminal constriction), the presence of granulation on legs IV, the presence of smooth lunules under the claws of leg IV, and the eggs with more numerous and well-defined areolae (9 surrounding each process with full areolation vs. 6 surrounding each process with semi-areolation), processes with long terminal ends, and fewer processes on the egg circumference (7–9 vs. 10–12), as well as by the PTP and mPTP analyses based on the currently available COI and 18S gene sequences (Fig. 1);
4. from *Mesobiotus hieronimi* (Pilato & Claxton, 1988)^46^ by the absence of eyes in living animals, the more posteriorly inserted stylet supports (*pt* 75.5–78.7 for the new species vs. *pt* 73.3–74.8), the shorter macroplacoids with different length sequence (2 < 1 < 3 vs. 2 = 3 = 1), the absence of leg granulation on legs I–III, and the eggs with more numerous areoles (9 vs. 6 surrounding each process) and generally shorter processes (18.5–28.7 μm vs. 25–34 μm) without divided apices;
5. from *Mesobiotus nuragicus* (Pilato & Sperlinga, 1975)^47^ by the absence of eyespots in living animals, the presence of longer third than first macroplacoids (macroplacoid length sequence 2 < 1 < 3 for the new species vs. 2 < 1 = 3), and the eggs with more numerous areoles (9 vs. 5–7 surrounding each process) delimited by reticulated ridges, longer processes (18.5–28.7 μm vs. up to 17 μm) typically without divided apices, and fewer processes on the egg circumference (7–9 vs. 12–13);
6. from *Mesobiotus pseudoliviae* (Pilato & Binda, 1996)^48^ by a smaller body length (225–400 μm for the new species vs. 600–800 μm), the absence of eyespots in living animals, the presence of longer third than first macroplacoids (macroplacoid length sequence 2 < 1 < 3 vs. 2 < 3 < 1), the smaller microplacoids (*pt* 8.4–10.8 vs. *pt* 12.0), the absence of leg granulation on legs I–III, the presence of smooth lunules under the claws of leg IV, and the smaller eggs (full diameter 81.6–99.6 vs. 156–177 μm) with fewer areolae surrounding each process (9 vs. 16) and much shorter (18.5–28.7 μm vs. 42–56 μm) and narrower (13.7–19.7 vs. 28–45 μm) processes;
7. from *M. skorackii* by the presence of longer third than first macroplacoids (macroplacoid length sequence 2 < 1 < 3 for the new species vs. 2 < 3 < 1), the absence of leg granulation on legs I–III, the presence of smooth lunules under the claws of leg IV, and the eggs with more numerous and well-defined areolae (9 surrounding each process with full areolation vs. 6 surrounding each process with semi-areolation), processes with long terminal ends and smaller base: height ratio (0.62–0.89 vs. 1.23–1.75), and fewer processes on the egg circumference (7–9 vs. 10–12), as well as by the PTP and mPTP analyses based on the currently available COI and 18S gene sequences (Fig. 1).

***Mesobiotus skanensis*** **sp. nov.**

**Fig. 10:**
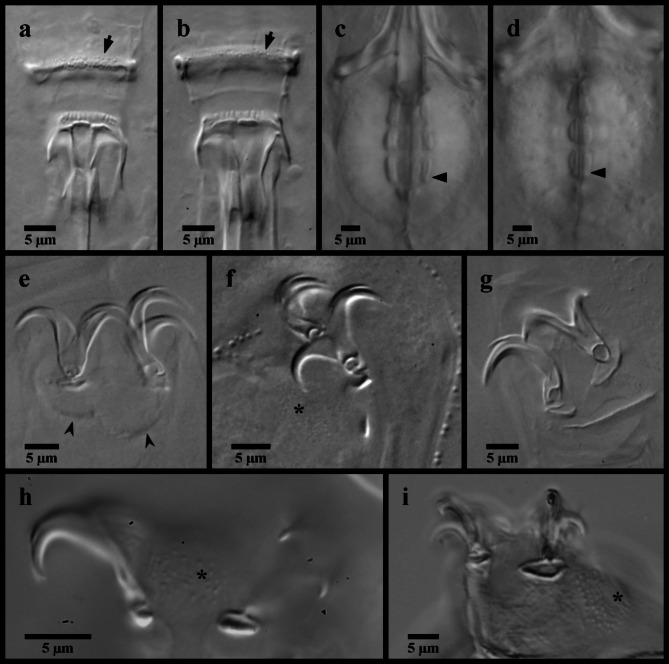
*Mesobiotus skanensis* sp. nov. (**a**,**e**,**f**,) photo of holotype specimen 22–163-1 in Hoyer’s medium, DIC; (**b**,**g**) paratype specimen 22–163-5 in Hoyer’s medium, DIC; (**c**,**d**) of live holoype specimen 22–163-1 in water, DIC; (**h**) specimen 22-140f in water, DIC (**i**) specimen 22-142f in water, DIC. (**a**) ventral oral cavity armature; (**b**) dorsal oral cavity armature; (**c**) placoid morphology, lateral view; (**d**) placoid morphology, dorsal view; (**e**) claws of leg I; (**f**,**h**) claws of leg II; (**g**,**i**) claws of leg IV. Black full arrows indicate first band of teeth; black flat arrowheads indicate the location of the constriction of the third macroplacoid; black indented arrowheads indicate the cuticular bars; the asterisk indicates granulation present on the legs.

**Fig. 11:**
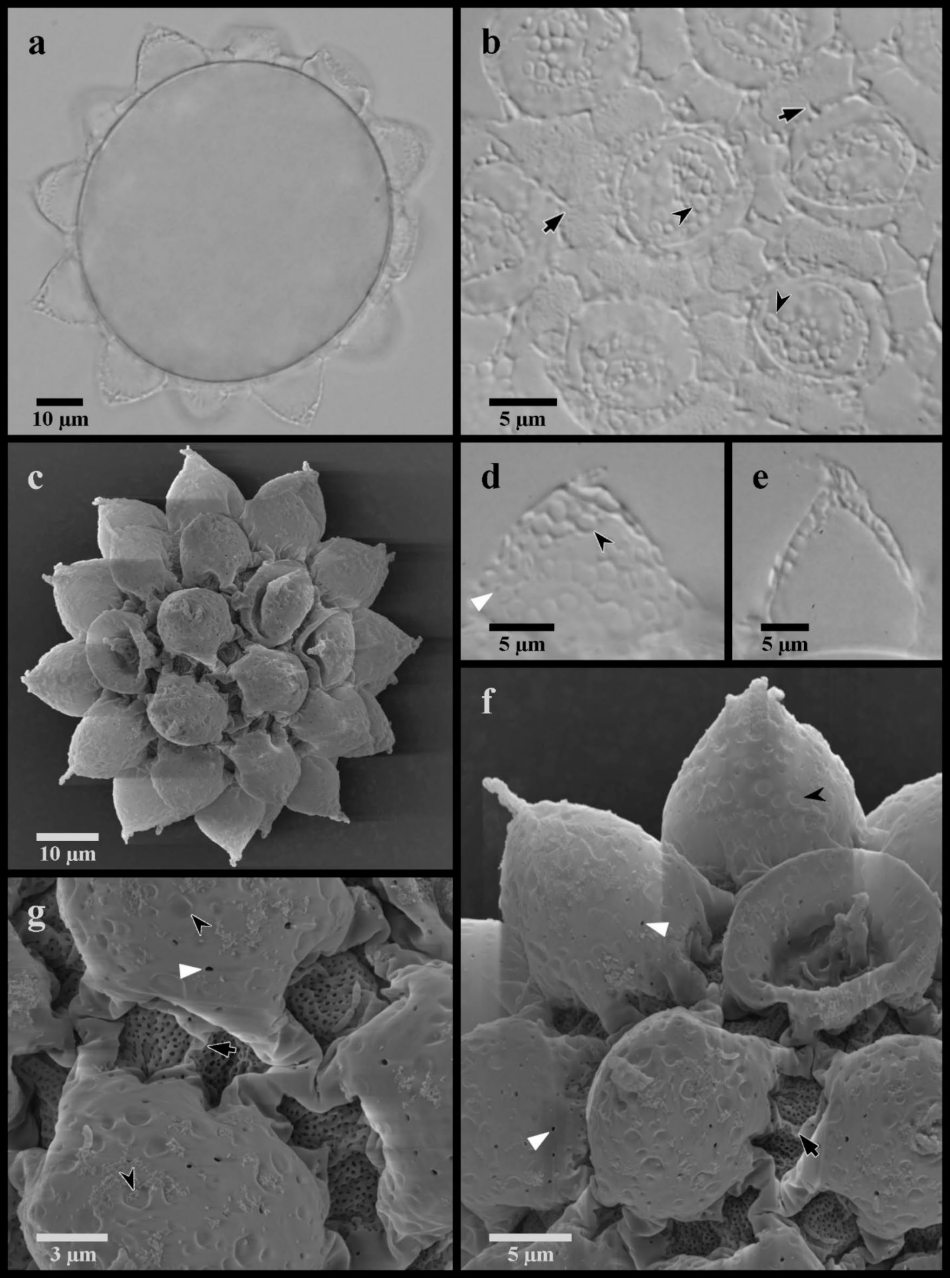
*Mesobiotus skanensis* sp. nov. eggs. (**a**) specimen 22–147-2, whole egg at the midsection; (**b**) specimen 22–147-4, egg surface; (**c**) SEM image of whole egg; (**d**) specimen 22–147-4, egg process; (**e**) specimen 22–147-1 egg processes; (**g**,**f**) SEM images of the egg processes and surface. Black full arrows indicate examples of the “finger-like projections” between the fully connected branches of the processes; black indented arrowheads indicate examples of larger cells that comprise the egg process reticulation; white flat arrowheads indicate examples of small pores present on the egg processes.

Figures 9, 10 and 11.

*Zoobank registration*. urn:lsid:zoobank.org:act:CF6EBA72-F4F4-4157-ABFB-BD455FD0AF45.

*Materials examined*. A total of twenty animals and seven eggs were analyzed and documented with digital video and micropictographs, including: ten animals and four eggs mounted on microscope slides in Hoyer’s medium; two eggs fixed on SEM stubs; and seven animals and one egg processed for DNA sequencing.

*Type Depositories*. The holotype (specimen 22–163-1; slide SMNH-Type-1560), five paratypes (specimens 22–163-2–5 and 24–090; slides SMNH-Type-1561–7), and four eggs (specimens 22–147-1–4; slides SMNH-Type-1568–70) are deposited at the Swedish National History Museum in Stockholm, Sweden. Four paratypes (specimens 22-116f, 22-117f, 22-118af and 22-118bf) are in the Bertolani Collection of University of Modena and Reggio Emilia (Italy).

*Type locality*. Sweden, Skåne, Gärds Köpinge, 55°57’12.2’’N 14°09’55.9’’E, moss on a stone wall by a small county road, collected on 20 May 2022 (Supplementary Fig. S5).

*Additional localities*.

- Sweden, Skåne, Gärds Köpinge, 55°57’12.2’’N 14°09’55.9’’E, moss on the bark of a European beech, taken at a height of ~ 1.5 m, collected on 20 May 2022;
- Sweden, Skåne, Forestad, 56°01’25.2’’N 13°20’51.9’’E, moss on a stone wall, collected on 17 April 2022.

*Diagnosis*. Species of *Mesobiotus*, 430–570 μm long, whitish to transparent in LM with small, black eyespots in living animals. OCA of *harmsworthi* type without accessory teeth. Macroplacoid length sequence 2 < 1 ≤ 3. Claws and lunules slightly smaller on leg I compared to legs II and III and slightly larger on leg IV. Lunules smooth. Eggs 91.2–106.6 μm full diameter, with 11–12 processes on the circumference. Processes in the shape of wide cones (base: height 0.84–1.36) with short, flexible tips; reticulated with meshes comprising numerous large cells evenly intermixed with fewer smaller cells. Proximal half of the process walls with small pores. Full areolation: ~6 large areolae surrounding each process, subdivided in two by an internal “finger-like projection”. Areolae surface with pores that appear as small dots in LM.

*Etymology*. The name *skanensis* refers to the type locality of Skåne, Sweden.

*Description*. Living animals transparent or whitish in LM with small black eyespots present in the lateral margins of the body at a level just anterior to the stylet supports (Fig. 9a). Eyespots less evident, although still present, after fixation in Hoyer’s medium. Body length 430–570 μm. Cuticle without sculpturing, gibbosities, spines, or pores, but with fine granulation present on the external surface of legs I–IV and surrounding the claws (Fig. 10f–i).

Bucco-pharyngeal apparatus of the *Macrobiotus* type, with terminal mouth and ten peribuccal lamellae.

OCA of *harmsworthi* type without supplementary teeth (Fig. 10a–b), comprising: (1) an anterior band of small granules at the base of the peribuccal lamellae, (2) a single posterior row of teeth in the shape of vertical ridges located just anterior to the third band of teeth and (3) three dorsal and three ventral transversal crests just before the buccal tube opening. Three transversal crests on the dorsal side shaped as bars of roughly equal size and width. Ventral crests thicker, with the two lateral crests situated slightly posterior to the medial crest.

Buccal tube rigid with long ventral lamina (30.0–37.9 μm, *pt* 65.7–71.1). Stylet supports inserted 34.8–43.0 μm (*pt* 77.5–80.7) from the anterior end of the buccal tube, leading to typically-shaped stylet system. Posterior end of the buccal tube within the pharyngeal bulb, terminating in a distinct thickening. Pharynx with triangular apophyses and rows of three macroplacoids and a single microplacoid (Fig. 10c–d). In frontal view, first macroplacoid shield-shaped, with a slightly rounded anterior end broadening to a triangular posterior end; second macroplacoid round; and third rectangular with an evident constriction ~ 3/4 of the way from the anterior end. Length of first, second and third macroplacoid 7.1–9.7 μm (*pt* 14.0–19.5), 5.0–8.2 μm (*pt* 11.3–15.4), and 7.8–9.7 μm (*pt* 14.6–19.4), respectively. Length sequence 2 < 1 ≤ 3. Microplacoids triangular, 4.1–5.9 μm (*pt* 8.6–12.4) long and situated closely posterior to the third macroplacoids.

Double-claws of the *Mesobiotus* type, with evident accessory points on the primary branch and lunules (Fig. 10e–i). Claws of the first pair of legs slightly smaller than those of the other legs; claws of the second and third pairs of legs about equal in length, and claws of the fourth pair of legs distinctly larger than the others, with the posterior claws larger than the anterior (Table 1). Lunules of all claws smooth. Divided cuticular bar present on each of the first three pairs of legs below the lunules (Fig. 10e).

Single ovary located dorsal-caudal to the intestine, occasionally with one or two well-developed eggs. Testes, seminal vesicles and seminal receptacles not seen.

Globular eggs laid freely, ornamented (Table 2; Fig. 11). Egg circumference with 11–12 processes (Fig. 11a) in the shape of wide cones terminating in a short flexible tip that is occasionally bifurcated. Processes 12.8–19.0 μm long and 12.4–17.9 μm wide at the base (base: height 0.84–1.36), reticulated in LM (Fig. 11b, d–e), with meshes comprising numerous, large circular cells (~ 1.2–2.2 μm) intermixed with a few smaller, circular cells (~ 0.4–0.8 μm) and dots. In SEM (Fig. 11c, f–g), the larger of these cells are visible as depressions, while the dots are revealed to be small pores scattered over the proximal half of each process. The bases of neighboring processes fully connect forming one complete areole between neighbors, and five or six complete areoles in total surrounding each process (Fig. 11b–c, f–g). “Finger-like projections”^14^ extend between the fully connected branches, occasionally forming partial connections that subdivide the full areole (Fig. 11b, f–g). The egg surface between the ridges (surface of the areolae) appears under LM with numerous small dots (Fig. 11b), which in SEM are shown to be numerous small pores (Fig. 11f–g).

*DNA Sequences*. Sequences from *M. skanensis* sp. nov. were attained for all three molecular markers and from seven adult animals and one egg. 28S and 18S were represented by a single haplotype, and COI was represented by two haplotypes (uncorrected p-value between COI haplotypes 0.76%):

- 18S: 1769 bp, GenBank accession number PQ367889;
- 28S: 921 bp, GenBank accession number PQ367895;
- COI haplotype-1: specimens 22–153, 22-140f – 142f, 22–154 and 22-247f; 658 bp; GenBank accession number PQ365777;
- COI haplotype-2: voucher 22–159 and 22–151; 658 bp, GenBank accession number PQ365778.

*Taxonomic Remarks*.

Circular or irregularly shaped patches of bumps or granules, ranging in size from ~ 0.5–1.4 μm in diameter, appeared present in patches on the ventral and dorsal body cuticle (Fig. 9b–d). However, since these bumps were not uniformly distributed or arranged in specific patterns and do not resemble body cuticle granulation commonly observed in other macrobiotids (e.g.^49–51^), their presence requires further confirmation with SEM analysis. The majority of the known species of *Mesobiotus *display a smooth body cuticle in LM (i.e. excluding body sculpturing visible only in SEM), with clearly evident tubercles, dots or granules reported from only three species (“very difficult to see” or “almost invisible” fine dots^49^ are reported from five additional species). The granules of *Mesobiotus pseudocoronatus *(Pilato, Binda & Lisi, 2006)^50^ are very small and distributed over the dorsal and lateral surfaces of the body. The granules of *Mesobiotus arguei *(Pilato & Sperlinga, 1975)^47^ and *Mesobiotus joenssoni *Guidetti, Gneuss, Cesari, Altiero & Schill, 2020^51^ are large (≥ 1 μm) and present only on the caudal extremity or posterior to the level of the third pair of legs, respectively.

The eggs of *M. skanensis* sp. nov. have 11–12 processes around the circumference that are shorter than 20 μm, reticulated, conical without divided apices, and that connect to their neighbors to form areolae. There are six other species of *Mesobiotus* with eggs with similar characteristics, and *M. skanensis* sp. nov. differs:

1. from *M. barbarae* by the presence of eyespots in living animals, the evident granulation on the first pair of legs, OCA with an undivided ventro-medial tooth in the third band, the longer ventral lamina (*pt* 65.7–71.1 for the new species vs. *pt* 58.2–63.2), the presence of smooth lunules under the claws of leg IV, and the eggs with more numerous (11–12 vs. 10) and shorter processes (height 12.8–18.0 μm vs. 18.4–26.5 μm);
2. from *Mesobiotus harmsworthi* (Murray, 1907)^52^, according to the redescription of Kaczmarek et al.^39^, by OCA without supplementary teeth around the second band of teeth, a larger microplacoid (*pt* 8.6–12.4 for the new species vs. *pt* 5.6–7.9), the presence of smooth lunules under the claws of the fourth pair of legs, and eggs always with well-defined areolae (full-areolation vs. semi-areolation) and processes that are reticulated with homogenous mesh sizes rather than evidently larger at the base and apex, as well as by the PTP and mPTP analyses based on the currently available COI and 18S sequences (Fig. 1);
3. from *M. hilariae* by OCA with *harmsworthi* type teeth (three bands of teeth visible in LM for the new species vs. posterior two bands only), the larger third than second macroplacoids (length sequence 2 < 1 ≤ 3 vs. 2 = 3 < 1), larger microplacoids (*pt* 8.6–12.4 vs. *pt 5.0–*8.3), the presence of smooth lunules under the claws of the fourth pair of legs, the smaller size of the claws of the fourth pair of legs (*pt* 26.4–34.6 vs. *pt* 34.3–45.9), and by the larger eggs (full diameter 91.2–106.6 vs. 71.3–89.1 μm) with areoles subdivided by “finger-like projections,” as well as by the PTP and mPTP analyses based on the currently available COI and 18S sequences (Fig. 1);
4. from *Mesobiotus neuquensis *Rossi, Claps & Ardohain, 2009^53^ by OCA without supplementary teeth between the second and third bands of teeth, a smaller pharyngeal bulb (42.5–53.3 μm for the new species vs. 62.0–69.4 μm), the evident granulation on all legs, and the much smaller eggs (bare diameter 61–78 μm vs. 87–94 μm) with more processes on the circumference (11–12 vs. 9–10) and process reticulation with much larger mesh sizes;
5. from *Mesobiotus ovostriatus *(Pilato & Patanè, 1998)^54^ by a larger body size (430–570 μm for the new species vs. 256–317 μm), OCA with *harmsworthi* type teeth (vs. *australis* type teeth *sensu* Kaczmarek et al.^14^), larger third macroplacoids (*pt* 14.6–19.4 vs. *pt* 12.2–13.9), larger microplacoids (*pt* 8.6–12.4 vs. *pt* 6.4–8.0), and eggs with differently shaped processes (process terminal tips short vs. long) with bases that lack the surrounding crown of dots and with areolaes subdivided by “finger-like projections”;
6. from *M. skorackii* by a larger ventral lamina (*pt* 65.7–71.1 vs. *pt* 61.1–64.9), larger third macroplacoids (*pt* 14.6–19.4 vs. *pt* 10.9–13.9), the presence of smooth lunules under the claws of the fourth pair of legs, and by the eggs with well-defined areolae (full-areolation vs. semi-areolation) subdivided by “finger-like projections” and processes that are reticulated with homogenous mesh sizes rather than increasing in size toward the apex, as well as by the mPTP and PTP analyses based on the currently available COI and 18S sequences (Fig. 1).

***Mesobiotus zelmae*** **sp. nov.**

**Fig. 12:**
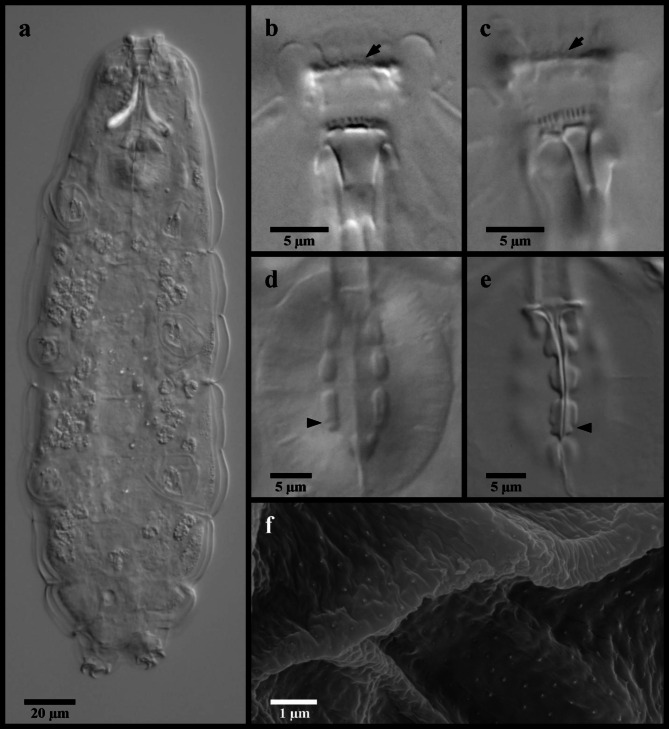
*Mesobiotus zelmae* sp. nov. (**a**) photo of live holotype specimen 22–215h in water; (**b**) specimen 22–250-15a fixed in Hoyer’s medium; (**c**,**e**) holotype specimen 22–215h in Hoyer’s medium; (**d**) specimen 22–239 in water. (**a**) whole body; (**b**) dorsal oral cavity armature; (**c**) ventral oral cavity armature; (**d**) placoid morphology, lateral view; (**e**) placoid morphology, dorsal view; (**f**) SEM image of the cuticle showing very fine granulation that is present over the entire body. Black full arrows indicate first band of teeth; black flat arrowheads indicate the location of the constriction of the third macroplacoid.

**Fig. 13:**
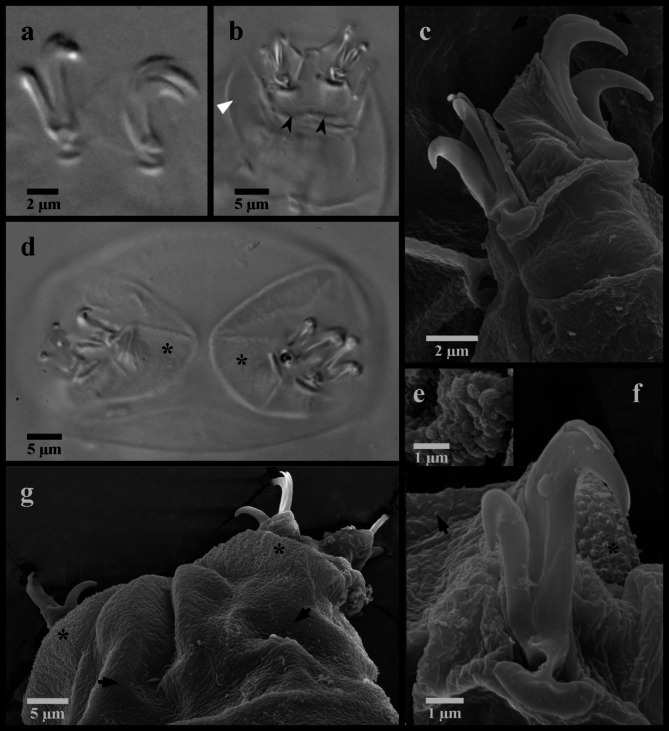
*Mesobiotus zelmae* sp. nov. claws. (**a**) specimen 22–250-15a, claws of leg I; (**b**) specimen 23–050, claws of leg II; (**c**) SEM image of the claws of leg II; (**d**) specimen 23–050, claws and legs IV; (**e**) SEM image of the large granules present on legs IV; (**f**) SEM image of the posterior claw of legs IV (**g**) SEM image of the claws and legs IV. Black full arrows indicate fine granules present over the entire body cuticle; black indented arrowheads indicate the cuticular bars; the white flat arrowhead indicates the cuticular bulge; asterisks indicate the large granulation present on legs IV.

**Fig. 14:**
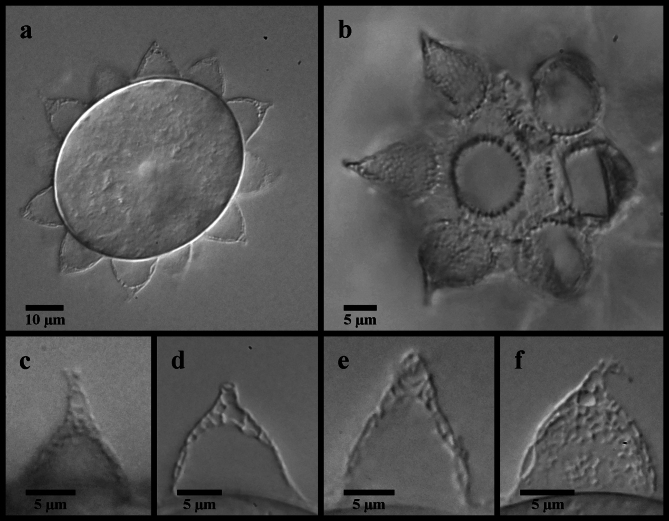
*Mesobiotus zelmae* sp. nov. eggs. (**a**,**b**,**f**) specimen 22–250-16; (**c**) specimen 22–449; (**d**,**e**) specimen 22–250-15e. (**a**) whole egg at the midsection; (**b**) surface of the egg and egg processes; (**c**,**d**,**e**,**f**) examples of egg processes.

Figures 12, 13 and 14.

*Zoobank registration*. urn:lsid:zoobank.org:act:1B217D6C-4E4F-4AF6-B573-B06221337FB0.

*Materials examined*. A total of 24 animals and seven eggs were analyzed and documented with digital video and micropictographs, including: seven animals and four eggs mounted on microscope slides in Hoyer’s medium; five animals fixed on SEM stubs; and twelve animals and three eggs processed for DNA sequencing.

*Type Depositories*. The holotype (specimen 22–215h; slide SMNH-Type-1571), three paratypes (24-356f, 24-366f and 24-367f; slides SMNH-Type-1572–4), and three eggs (specimens 22–052, 22–250-15e and 22–250-16; slides SMNH-Type-1575–7) are deposited at the Swedish Natural History Museum in Stockholm, Sweden. Three paratypes (specimens 24-258f, 24-259f and 24-370f) and one egg (specimen 22–449) are in the Bertolani Collection of University of Modena and Reggio Emilia (Italy).

*Type locality*. Sweden, Skåne, Forestad. 56°01’25.2’’N 13°20’51.9’’E, moss on a stone wall, collected 17 April 2022 (Supplementary Fig. S6);

*Additional localities*.

- Sweden, Skåne, Fjälkestad, 56°08’58.3’’N 14°11’46.5’’E, one sample of spruce needle litter, one sample of moss from the bark of a spruce tree taken from a height of ~ 1.5 m, one sample of moss on a rock, collected on 23 March 2022;
- Sweden, Skåne, Fjälkestad, 56°08’58.7’’N 14°11’53.2’’E, moss on a rock, collected on 23 March 2022;
- Sweden, Skåne, Forestad. 56°01’25.2’’N 13°20’51.9’’E, moss on a stone wall, collected 17 April 2022;
- Sweden, Skåne, Bockeboda recreational area, 56°01’53.9’’N 13°59’30.4’’E, leaf litter primarily from common hazel, collected 21 March 2022;
- Sweden, Skåne, Simrishamn, 55°38’43.8’’N 14°11’30.1’’E, leaf litter primarily from European beech and moss on a rock, collected 20 May 2022.

*Diagnosis*: Species of *Mesobiotus* without eye-spots, 215–395 μm long. Body cuticle smooth with evident granulation on legs I–IV. OCA of *harmsworthi* type without accessory teeth. Pharyngeal bulb with rows of three macroplacoids and closely situated microplacoid. Macroplacoid length sequence 2 < 1 < 3. Lunules under claws on legs I–IV smooth. Eggs 71.7–81.3 μm full diameter, with 10–12 processes on the circumference. Each egg process cone-shaped (base: height 0.67–0.98) with a short terminal tip, appearing finely reticulated in LM, with crown of thickenings at the base. Egg surface between processes wrinkled and sparsely dotted.

*Etymology*. Named for Zelma Bates, daughter of the first author, who helped collect these tardigrades while also learning to walk.

*Description*. Living animals 215–395 μm in length, transparent or whiteish in LM (Fig. 12a) and transparent after fixation in Hoyer’s medium. Eyespots absent in living animals. Body cuticle smooth in LM without sculpturing, gibbosities, spines, papillae or pores. In SEM, very small granules visible regularly distributed over the entire body cuticle (Figs. 12f and 13g). Granulation on legs I-IV visible in LM and SEM, with fine granules on the external surfaces of legs I–III and larger granules present on the dorsal and lateral sides of legs IV (Fig. 13d–g). A flattish cuticular bulge (pulvinus) present on the internal surface of legs I-III (Fig. 13b).

Bucco-pharyngeal apparatus of the *Macrobiotus* type, with terminal mouth and ten peribuccal lamellae.

OCA of *harmsworthi* type without supplementary teeth (Fig. 12b–c), comprising: (1) an anterior field of fine granules at the base of the peribuccal lamellae that can be difficult to see in smaller individuals, (2) a single posterior row of teeth in the shape of vertical ridges located just anterior to the third band of teeth, and (3) three each dorsal ventral transversal crests just before the buccal tube opening. Transversal crests on the dorsal side shaped as bars, with the paired lateral crests approximately equal in width to the medial crest. Ventral crests comprising rectangular-shaped medial crest and triangular paired lateral crests.

Rigid buccal tube with ventral lamina 17.3–25.6 μm (*pt* 56.9–71.1) long and stylet supports inserted 21.5–31.2 μm (*pt* 74.7–79.5) from the anterior end. Pharyngeal bulb with triangular apophyses and rows of three macroplacoids and single microplacoid (Fig. 12d–e). In frontal view, first macroplacoid arrow-shaped with a slight narrowing at the midpoint; second rounded; and third oblong with subterminal constriction leading to a rounded and distinctly narrower posterior end. Length of first, second and third macroplacoids 3.7–5.7 μm (*pt* 12.5–14.8), 3.1–4.9 μm (*pt* 10.5–13.0), and 3.7–6.1 μm (*pt* 13.6–15.9), respectively. Length sequence 2 < 1 < 3. Microplacoids relatively large and triangular, 2.9–4.5 μm (*pt* 9.9–13.0) long and situated closely posterior to the third macroplacoids.

Double-claws of the *Mesobiotus* type, with evident accessory points on the primary branch (Fig. 13). Claws and lunules of legs I–III approximately equal in length; claws and lunules of legs IV larger (Table 1). Lunules under claws of legs I–IV smooth. A continuous cuticular bar present on each of the first three pairs of legs below the lunules (Fig. 13b).

Gonochoristic. Male specimens with small testis with spermatozoa and female specimens with ovary with large eggs found. Seminal receptacle not seen. No morphological secondary sexual dimorphism such as male gibbosities was identified.

Globular eggs laid freely, ornamented, 71.7–81.3 μm in diameter including processes (Table 2; Fig. 14). Egg circumference with 10–12 processes (Fig. 14a). Each process cone-shaped with a short, pointed tip (Fig. 14c–f); 11.8–15.8 μm long and 10.2–14.6 μm in diameter at the base (base: height 0.67–0.98). Tips occasionally bifurcated, but more typically undivided. Process walls appear finely reticulated in LM with meshes comprising small cells evenly distributed over the entire process. Larger “bubbles” in terminus present only in longer processes. Process base with crown of thickenings (Fig. 14b), unconnected to neighboring processes (e.g. full or partial areoles not present). Egg chorion in between sparsely dotted and wrinkled.

*DNA Sequences*. Sequences from the *M. zelmae* sp. nov. were attained for all three molecular markers, including from twelve adult animals and two eggs. 28S was represented by a single haplotype, 18S was represented by two haplotypes (uncorrected p-value between 18S haplotypes 0.06%) and COI was represented by four haplotypes (range of uncorrected p-value between COI haplotypes 0.30–1.57%):

- 18S haplotype-1: specimens 22–239 and all others excluding 22–115; 1769 bp, GenBank accession number PQ367890;
- 18S haplotype-2: specimen 22–115; 1769 bp, GenBank accession number PQ367891;
- 28S: 921 bp, GenBank accession number PQ367896;
- COI haplotype-1: specimens 22–239, 22–214, 22–217, 22–218, 22–233 and 22–244; 658 bp; GenBank accession number PQ365779;
- COI haplotype-2: specimens 23–050, 22–160; 22–207; 22–405 and 22-442f; 658 bp, GenBank accession number PQ365780;
- COI haplotype-3: specimen 22–115; 658 bp; GenBank accession number PQ365781;
- COI haplotype-4: specimens 22–215 and 210; 658 bp; GenBank accession number PQ365782.

*Taxonomic Remarks. Mesobiotus zelmae* sp. nov. was present in the majority (65%) of the samples collected in this study, reflective of a potentially widespread distribution in southern Sweden, and was collected alongside *M. skanensis* sp. nov., *M. emiliae* and, most frequently, *M. mandalori* (Table 3). While the adults and eggs of *M. zelmae* sp. nov. are relatively easily distinguishable from *M. skanensis* sp. nov. (see below), the adults and eggs of *M. zelmae* sp. nov., *M. mandalori* and *M. emiliae* differ in few characters and, as with *M. bockebodicus* sp. nov., they should be carefully considered before morphological identification. That being stated, results from the phylogenetic, PTP and mPTP analyses (Fig. 1) clearly support *M. zelmae* sp. nov. as a separate species, and it can be distinguished morphologically from all other species collected in this study. Specifically, *M. zelmae* sp. nov. differs:

**Table 3:** Location within Skåne County, Latitudinal and longitudinal coordinates, and substrate/habitat information of each sample examined for this study. Occurrences of adult and egg specimens are marked: T = type localities, X = occurrences at locations other than the type locality, *=DNA sequences were attained from one or more specimens from this sample. ASL above sea level.

Locality	Coordinates	Substrate/Habitat	Sample	*M. emiliae*		*M. mandalori*		*M. skanensis* sp. nov.		*M. bockebodicus* sp. nov.		*M. zelmae* sp. nov.	
				Adult	Egg	Adult	Egg	Adult	Egg	Adult	Egg	Adult	Egg
Bockeboda	56°01’38.7’’N 13°59’33.9’’E	Moss on a rock; altitude 76 m ASL	8a	X*	X					X*	X		
Bockeboda	56°01’38.7’’N 13°59’33.9’’E	Leaf litter from beech; altitude 76 m ASL	8b							T*	T*		
Bockeboda	56°01’53.9’’N 13°59’30.4’’E	Leaf litter from hazel; altitude 78 m ASL	16			X*						X*	
Fjälkestad	56°08’58.7’’N 14°11’53.2’’E	Moss on a rock; altitude 42 m ASL	18									X*	
Fjälkestad	56°08’58.3’’N 14°11’46.5’’E	Leaf litter from spruce; altitude 45 m ASL	20a			X*						X*	X
Fjälkestad	56°08’58.3’’N 14°11’46.5’’E	Moss on a rock; altitude 45 m ASL	20b		X	X*	X					X*	
Fjälkestad	56°08’58.3’’N 14°11’46.5’’E	Moss on the bark of a spruce tree, taken from ~ 1.5 m up; altitude 45 m ASL	20c				X					X*	X
Klippan	56°01’25.2’’N 13°20’51.9’’E	Moss on a stone wall; altitude 50 m ASL	59			X*		X*	X			T*	T*
Anderstorp	56°00’01.3’’N 13°19’31.4’’E	Moss on the metal rail separating the road and a juniper stand; altitude 70 m ASL	66									X*	X
Simrishamn	55°38’43.8’’N 14°11’30.1’’E	Moss on a rock; altitude 133 m ASL	136a									X*	
Simrishamn	55°38’43.8’’N 14°11’30.1’’E	Leaf litter from beech; altitude 133 m ASL	136b			X*							X*
Gärds Köpinge	55°57’12.2’’N 14°09’55.9’’E	Moss on the bark of a beech tree, taken from ~ 1.5 m up; altitude 11 m ASL	138a					X*					
Gärds Köpinge	55°57’12.2’’N 14°09’55.9’’E	Moss on a stone wall by a small county road; altitude 11 m ASL	138b					T*	T*				
Sånnarna	55°55’44.2’’N 14°14’46.7’’E	Moss on sandy soil; altitude 9 m ASL	SHH	T*	T*

1. from *M. emiliae* by the granulation on legs I–III (visible in LM), the differing macroplacoid length sequence (2 < 1 < 3 vs. 1 < 2 < 3), the presence of cuticular bars under the claws of legs I–III, and the eggs with taller processes (11.8–15.8 μm vs. 7.9–10.6 μm) that are always longer than the diameter of the base (base: height 0.67–0.98 vs. 1.44–1.64) and with larger interprocess distances (2.6–3.7 μm vs. 0.5–1.6 μm);
2. from *M. mandalori* by the lack of eyespots in living animals, wider buccal tube (external width *pt* 16.1–19.1 for *M. zelmae* sp. nov. vs. *pt* 11.8–14.7), the differing macroplacoid length sequence (2 < 1 < 3 vs. 1 < 2 < 3), the smooth lunules under the claws of legs IV, and the smaller eggs (bare diameter 46.1–51.9 μm vs. 53.7–77.7 μm) with processes that are always longer than the diameter of the base (base: height 0.67–0.95 vs. 1.06–1.60);
3. from *M. bockebodicus* sp. nov. by the absence of brown coloring in living adults, the presence of evident granulation on legs I–III, and the smaller eggs (full diameter 71.7–81.3 μm for *M. zelmae* sp. nov. vs. 81.6–99.6 μm) with more numerous (10–12 v. 7–9 on the circumference) and shorter processes (11.8–15.8 μm vs. 18.5–28.7 μm) that each terminate in a short flexible tip (vs. a long, slender ending) and that do not connect to neighboring processes (process base a crown of thickenings vs. full areolation);
4. from *M. skanensis* sp. nov. by the smaller body size (215–395 μm for *M. zelmae* sp. nov. vs. 430–570 μm), lack of eyespots, as well as by the smaller eggs (full diameter 71.7–81.3 μm vs. 91.2–106.6 μm) with processes with a basal crown of thickenings (vs. full areolation);

In addition, *Mesobiotus zelmae* sp. nov. is morphologically very similar to three other species of *Mesobiotus*, sharing the following characters: *harmsworthi* type OCA, a smooth body cuticle under LM, smooth lunules under all claws, and eggs with 12 or fewer cone-shaped processes on the circumference that terminate in a short tip, with processes that are longer than the diameter of their base (process base: height ratio < 1.00) and without areolae between processes. However, *M. zelmae* sp. nov. can be distinguished:

1. from *Mesobiotus baltatus *(McInnes, 1991)^55^ by the absence of brown bands and eyespots in living animals, a wider buccal tube (*pt* 16.1–19.1 for the new species vs. *pt* 13–16), larger third than first macroplacoids (macroplacoid length sequence 2 < 1 < 3 vs. 2 < 3 ≤ 1), smaller second macroplacoids (3.1–4.9 μm vs. 5.5 μm), and by the smaller eggs (full diameter 71.7–81.3 μm vs. 91–116 μm) with processes with a basal crown of ridges;
2. from *Mesobiotus patiens* (Pilato, Binda, Napolitano & Moncada, 2000)^56^ by the different shapes of the first and third macroplacoids (anterior half of the first macroplacoids narrower and concave and the posterior end of the third macroplacoids narrower and more rounded for the new species), larger third than first macroplacoids (macroplacoid length sequence 2 < 1 < 3 vs. 2 < 3 < 1), and by smaller eggs (full diameter 71.7–81.3 μm vs. 90.5–100 μm) with processes that are positioned more distantly from each other;
3. from *Mesobiotus reinhardti* (Michalczyk & Kaczmarek, 2003)^57^ by the smaller body size (215–395 μm for the new species vs. 399–631 μm), wider buccal tube (*pt* 16.1–19.1 vs. *pt* 10.4–14.9), OCA without supplementary teeth, larger microplacoids (*pt* 9.9–13.0 vs. *pt* 6.9–9.8), and by the much smaller eggs (full diameter 71.7–81.3 μm vs. 123.5–147.3 μm) with smaller processes (11.0–15.8 μm vs. 20.0–27.2 μm) without the large bubble-like meshes (in LM) in the upper part.

## Discussion

The use of nucleotide sequences to delineate species and capture biodiversity is an important tool for advancement in the study of tardigrades. It is important for *Mesobiotus *taxonomy since the relatively low morphological interspecific variation between the adults of the genus means that correct species identification from morphology alone requires numerous measurements of minute details of the animals as well as knowledge of the morphology of their eggs^14^. This involves high amounts of effort, specimens and specialized expertise and is further complicated by the fact that multiple, different species of *Mesobiotus* can be present in a single sample. Of the fourteen samples examined in this study, half contained two or more species of *Mesobiotus* (Table 3), and instances of sympatric species have similarly been reported from e.g. Norway^6^, Svalbard^39^, and Vietnam^38^. Notably, all species described herein can be distinguished through differences in mitochondrial and ribosomal nucleotide sequences as well as morphologically, i.e. they are not cryptic species. Further, animals and egg specimens of the same species were morphologically consistent across sampling locations and were confirmed by DNA analysis (Table 3). Such findings suggest that collecting multiple species from the same place is not just possible but likely and belies the assumption that an egg and an adult are of the same species simply because they were simultaneously collected. In one of the examined samples (Table 3), all animals collected were of a different species than that of all eggs from the same sample. Alternately, sympatry cannot necessarily be assumed when different eggs from the same sample are collected since instances of extreme intraspecific egg variation have been documented (e.g^58–60^). Attaining molecular data from both adults and eggs can be utilized in lieu of culturing to confirm the egg morphology of a species and generally provides a robust, reproducible and relatively easy method of species identification that is also useful for the morphological association between animal and egg found in the field.

The morphological analyses, particularly the ultrastructural investigations with SEM, also provided important information about the genus. The very small granules visible only with SEM that were regularly distributed over the entire body cuticle of *M. zelmae* and *M. emiliae* (Figs. 2d and 12f) have similarly been observed in *Mesobiotus occultatus *Kaczmarek, Zawierucha, Buda, Stec, Gawlak, Michalczyk & Roszkowska 2018^39^, *Mesobiotus anastasiae *Tumanov, 2020^21^ and *Mesobiotus philippinicus *Mapalo, Stec, Mirano-Bascos & Michalczyk, 2016^61^. As these granules are visible only with SEM, their presence was most probably overlooked in other *Mesobiotus* species descriptions and potentially could represent a feature of the genus. Similar cuticular granules have also been found in species of *Macrobiotus*^16,62^ and *Xerobiotus*^8^, which indicates that this character may be widespread within Macrobiotoidea.

Many tardigradologists have adopted an integrated molecular and morphological approach when introducing new species of *Mesobiotus*(e.g^15,16,21,37,38^). and are simultaneously filling in the knowledge gap by providing new DNA sequences for species previously described (e.g^16,63^). Including the three new species and *M. emiliae*, 31 species of the genus (38%) now have genetic data available. Nevertheless, this represents only a small fraction of what is required to understand the true biodiversity and evolutionary history of the genus. Results from the ML analyses mirrored previous findings, showing that all non-Antarctic *Mesobiotus *formed a monophyletic clade that was separate from their Antarctic counterparts^16,18,63^ and was subdivided into three well-supported subclades. However, there was no evident morphological, ecological or geographic trait that united one subclade to the exclusion of the others in our analyses, and relationships within the subclades remain unclear.

Results from this study once again confirmed that the two former species complexes from which *Mesobiotus* was established, the *harmsworthi* and *furciger *groups, are non-monophyletic, and thus, the morphological similarities of the egg ornamentations on which the morpho-groups are based^16^ are analogous. Unlike most genera of Macrobiotidae, the different egg morphologies are non-uniform within *Mesobiotus* with even closely related species showing different egg processes. For example, within the small phylogenetic cluster comprising *M. bockebodicus* sp. nov. (Fig. 1), two different morpho-groups and three different egg process morphologies were present: *M. bockebodicus* sp. nov. (*harmsworthi* morpho-group) with processes in shape of “cones with long slender ending and filaments” with basal “full areolation”, *M. emiliae* (*harmsworthi* morpho-group) with “wide sharp cones” showing a basal “crown of thickening”, and *Mesobiotus peterseni* (Maucci, 1991)^64^ (*montanus* morpho-group) with dome-shaped processes with small “finger-like projections” at the base (definitions according to Kaczmarek et al.^14^). The value of informal morpho-groups to taxonomists and systematists has recently been debated, with those opposed arguing that continued use causes confusion and will distract from the pursuit of true evolutionary relationships and biogeographical patterns^19^, while those in favor believe that informal groups allow for better identification and communication of the taxa and postulate that any confusion should be alleviated with better defined morpho-groups^16^. As all three new species presented in this study belong to the informal *harmsworthi* morpho-group, that group now includes 80% (66/82) of the known species of *Mesobiotus* (the *furciger* and *montanus* morpho-groups comprise 11 and 5 nominal species, respectively). Thus, the three morpho-groups are so disproportionate in size that there seems very little benefit to their continued use, or at least little benefit to the continued use of the *harmsworthi* morpho-group as it currently stands. That is, it is just as easy to compare taxa of the *harmsworthi* group to all species of *Mesobiotus* than it is to restrict such contextualization to “only” the 66 species with similar eggs, and doing so does not give the impression that egg characteristics are the most important feature separating the genus, something the evolutionary relationships within *Mesobiotus* as indicated by all modern phylogenetic analyses do not support.

Finally, with the introduction of the three new species from southern Sweden and the new record of *M. mandalori*, the total number of known species of Tardigrada and Macrobiotidae in the country increases to 120 and 26, respectively, of which 62 (52%) and 19 (73%) occur in Skåne County and 37 (31%) and 15 (57%) occur specifically in the KVBR. Indeed, *M. zelmae* sp. nov. and *M. mandalori* are particularly noteworthy not only because they were sympatric with each other as well as *M. emiliae* but because their distributions were quite widespread in the KVBR area (Table 3). Combining our results with those of Massa et al.^22^and the fact that tardigrade species abundance is directly related to sampling effort^65^, it is almost certain that additional sampling in this tardigrade hotspot will reveal even more hidden diversity.

## Methods

### Collection and extraction of samples

Fourteen samples of mosses, leaf litter, and soil from eight sampling areas were collected from March–May in 2022 within Skåne (Table 3). All collection permissions were obtained where required and this field study did not involve endangered or protected species. Samples were stored in plastic bags or glass jars and transported back to the Swedish Natural History Museum in Stockholm, where they were allowed to air dry at room temperature. Samples were stored at room temperature for up to eighteen months before further processing.

Samples were rehydrated in tap water for 30–60 min and then animals and eggs were extracted using sieves of mesh size 250 and 40 μm. Specimens were manually isolated from the extraction under a Nikon SMZ 1500 stereomicroscope and transferred to a glass slide and identified with a Nikon Eclipse 80i compound microscope equipped with differential interference contrast (DIC). Light micrographs and digital videos were captured with a Canon EOS 5D Mark III digital camera. Specimens were initially placed in enough water that the animal was very lightly pressed but could still move relatively freely. Observations and photographs/videos were made for ~ 1–2 min, after which water was slowly removed and the specimen progressively squeezed in order to better visualize and document finer details and structures. Specimens were observed and documented using a 100X immersion oil objective. Measurements were taken with an ocular micrometer from live animals and animals preserved in Hoyer’s medium and from photographs of live animals using GMP v 2.10.

Following documentation, individual specimens were either remounted on slides in Hoyer’s medium for further observations, fixed in 95% ethanol for subsequent DNA extraction, or prepared for scanning electron microscope (SEM) observations according to the techniques of Guidetti et al.^31^ and viewed with FEI Quanta FEG 650 SEM at the Swedish Natural History Museum. A specimen of *M. emiliae*, previously prepared for SEM by Massa et al.^22^, was observed with a Nova Nano SEM 450, FEI company at the “Centro Interdipartimentale Grandi Strumenti”, University of Modena and Reggio Emilia (Italy).

### Morphometric measurements

Measurements were taken following the guidelines of Pilato^66^and Kaczmarek & Michalczyk^67^. Measurements of specimens fixed in Hoyer’s medium were taken following fixation. The body length of the living animals was measured excluding the hind legs, and the buccal tube length was measured from the anterior end at the level of the stylet sheath to the posterior end within the pharynx. To ensure proper levelling^68^, the buccal tube was only measured when both anterior and posterior ends were on the same focal plane. Placoids and placoid rows were measured in lateral view. Claws were only measured when correctly positioned in full frontal or lateral view. For eggs that were oval, the widest diameter was reported. Egg processes height was only measured in full lateral view on the egg circumference. Raw morphometric data are given in Supplementary Tables S2–S4 and were handled using the Parachela ver. 1.8 template available from the Tardigrada Register, www.tardigrada.net/register^69^. All figures were assembled in GIMP 2.10.30. For structures that could not be fully focused in a single photograph, a series of 2–5 images were taken and manually assembled into a single stacked image with deep focus.

### Extraction of DNA and molecular analyses

DNA was extracted from whole animals or eggs using the DNeasy Blood & Tissue kit (Qiagen) following the manufacturer’s instructions. PCR amplification was performed using 0.2 ml PuReTaq Ready-to-go PCR Beads (GE Healthcare) with 5 pmol each forward and reverse primers and 3 µl DNA. Three gene fragments were selected for DNA amplification and analyses: the complete nuclear small ribosome subunit (18S) gene, a ~ 900 bp region of the large ribosome subunit (28S) gene and the ~ 650 bp segment of the cytochrome oxidase c subunit I (COI) gene pertaining to the “Folmer region”^70^. In order to amplify the entire 18S locus, three primer sets amplifying three partially overlapping fragments were utilized. The overlapping portions of the fragments were aligned in order to combine the three fragments and acquire the full 18S gene sequence. Supplementary Table S5lists the primer pairs and protocols used for amplification and sequencing. Amplification of a fourth gene locus, the internal transcribed spacer (ITS-2), was attempted but was unsuccessful for the specimens in this study (no amplicons occurred). Products were viewed on a 1% agarose gel, purified using ExoSAP-IT enzymes (Exonuclease and Shrimp Alkaline Phosphotase; GE Healthcare) and sent to Macrogen Europe (Netherlands) for commercial sequencing. Sequence assembly was performed in Mega v. 7.0.21^71^.

All available and published sequences for all species of *Mesobiotus* as well as *Paramacrobiotus tonollii *(Ramazzotti, 1956)^72^, *Tenuibiotus tenuiformis *(Tumanov, 2007)^73^, *Sisubiotus hakaiensis *Vecchi, Choong & Calhim, 2022^74^, *Macrobiotus macrocalix *Bertolani & Rebecchi, 1993^75^ and *Xerobiotus gretae *Massa, Guidetti, Cesari, Rebecchi & Jönsson, 2021^22^, which were selected to represent the outgroup, were downloaded from GenBank for each locus and combined with the new sequences (Supplementary Table S6). Sequences were aligned with Multiple Alignment using Fast Fourier Transformation (MAFFT^76^;), with COI sequences first translated into amino acids using the standard invertebrate mitochondrial genetic code, manually checked for stop codons and reading frame shifts and reverted to the original nucleotides. To account for poorly aligned positions or regions saturated by multiple substitutions, 18S alignments were additionally filtered with Gblocks 0.91b allowing for smaller blocks and gap positions for less stringent selection of the final blocks, although all analyses were performed using both filtered and nonfiltered alignments.

Maximum likelihood (ML) analysis was performed on each marker individually as well as with two concatenated datasets: the first with all three above-mentioned gene regions, and the second with only the 18S and COI loci since the majority of the species of *Mesobiotus*for which any genetic data is currently available never-the-less lack sequence data from a comparable region of the 28S gene. All ML analyses were performed in IQTree v. 2.3.2^77^with 1000 ultrafast bootstrap replicates^78^. ModelFinder^79^ via BIC as implemented in the IQTree software determined the best fitting substitution models for each dataset: TIM2e + I + G4 for 18S and 28S and GTR + F + I + G4 for COI.

Species were inferred using the multi-rate and single-rate Poisson tree process (mPTP and PTP, respectively) under ML and Markov chain Monte Carlo (http://mptp.h-its.org^80^;). Both analyses were run using input trees generated from ML analyses of COI and COI-18S concatenated datasets. Following recommended guidelines^80^, outgroup sequences were excluded from the PTP and mPTP analyses. Uncorrected pairwise distances (p-distances) was calculated in MEGA X^71^.

## Electronic supplementary material

Below is the link to the electronic supplementary material.

Supplementary Material 1

Supplementary Material 2

Supplementary Material 3

Supplementary Material 4

Supplementary Material 5

Supplementary Material 6

Supplementary Material 7

Supplementary Material 8

Supplementary Material 9

Supplementary Material 10

## Data Availability

The datasets analyzed during the current study are available in the GenBank repository, with all accession numbers and voucher information listed in Supplementary Table S6. All type materials were deposited at the Swedish Natural History Museum in Stockholm, Sweden or at the Bertolani Collection of University of Modena and Reggio Emilia, Italy.
