# Supplementary figures and images for: Three new species of Mesobiotus (Eutardigrada: Macrobiotidae) from Sweden with an updated phylogeny of the genus
Source: Sci Rep. 2025 Feb 6;15:4535. doi: 10.1038/s41598-025-88063-8 (PMC11802830; doi:10.1038/s41598-025-88063-8)

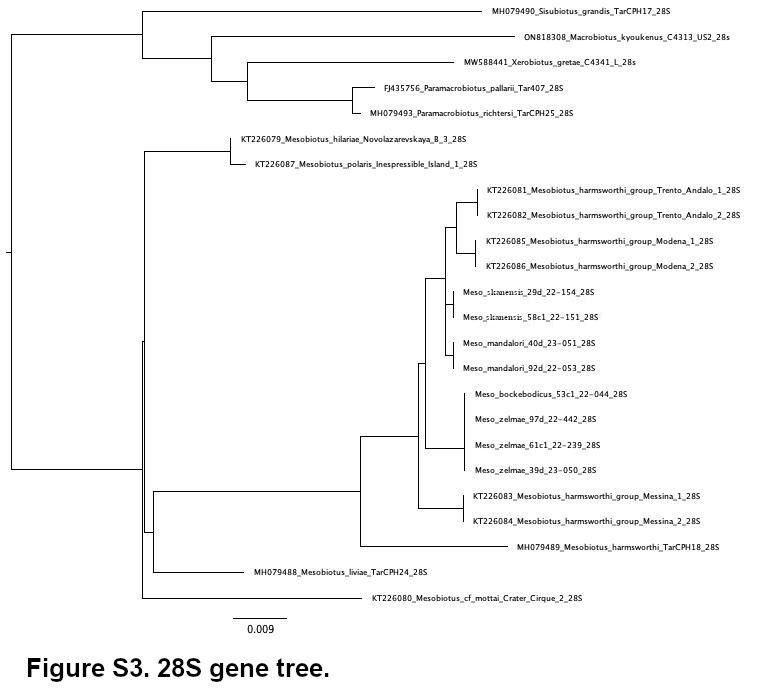

Supplement: Supplementary file 1 — Supplementary Material 1 [file 41598_2025_88063_MOESM1_ESM.jpeg]

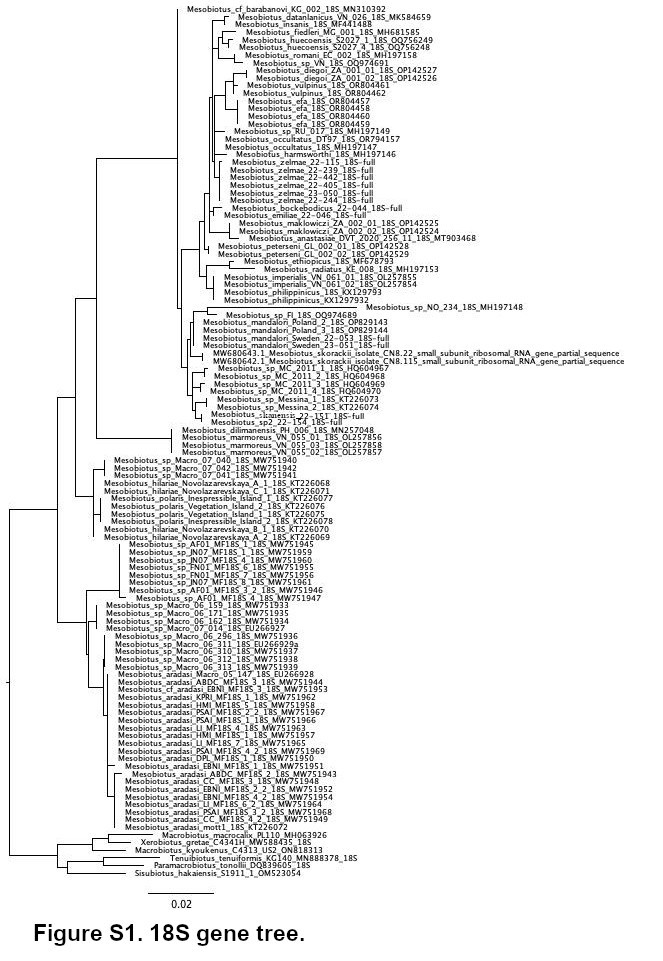

Supplement: Supplementary file 2 — Supplementary Material 2 [file 41598_2025_88063_MOESM2_ESM.jpeg]

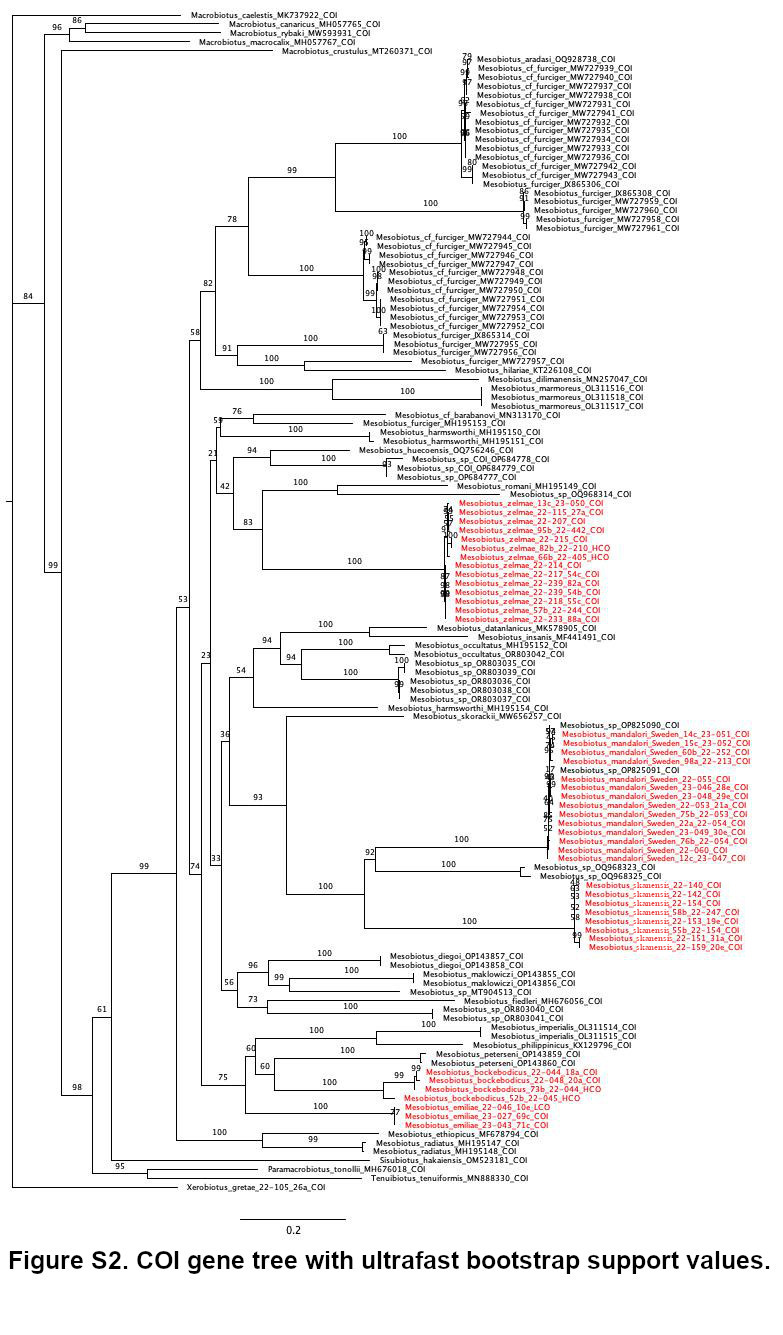

Supplement: Supplementary file 7 — Supplementary Material 7 [file 41598_2025_88063_MOESM7_ESM.jpeg]

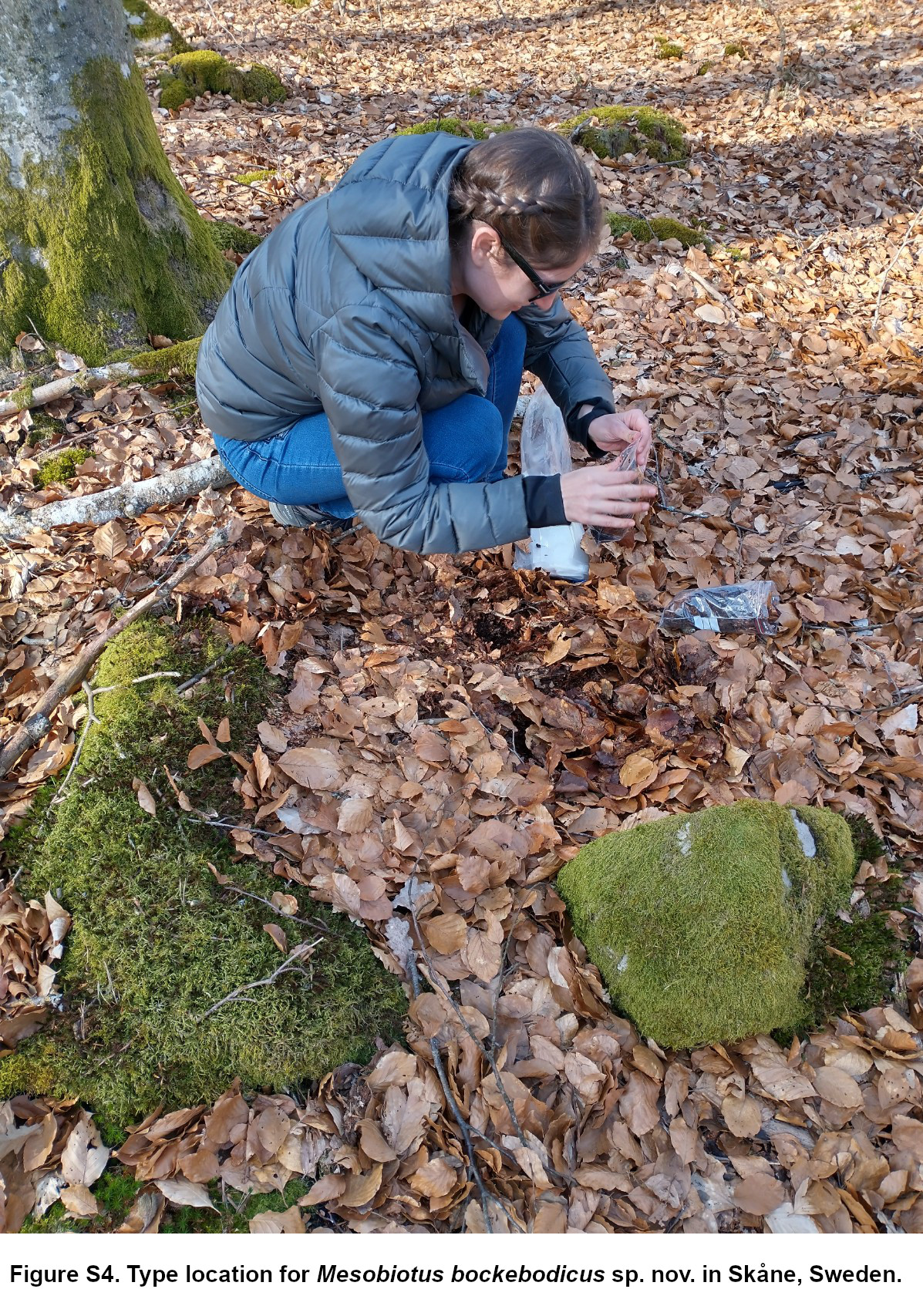

Supplement: Supplementary file 8 — Supplementary Material 8 [file 41598_2025_88063_MOESM8_ESM.jpeg]

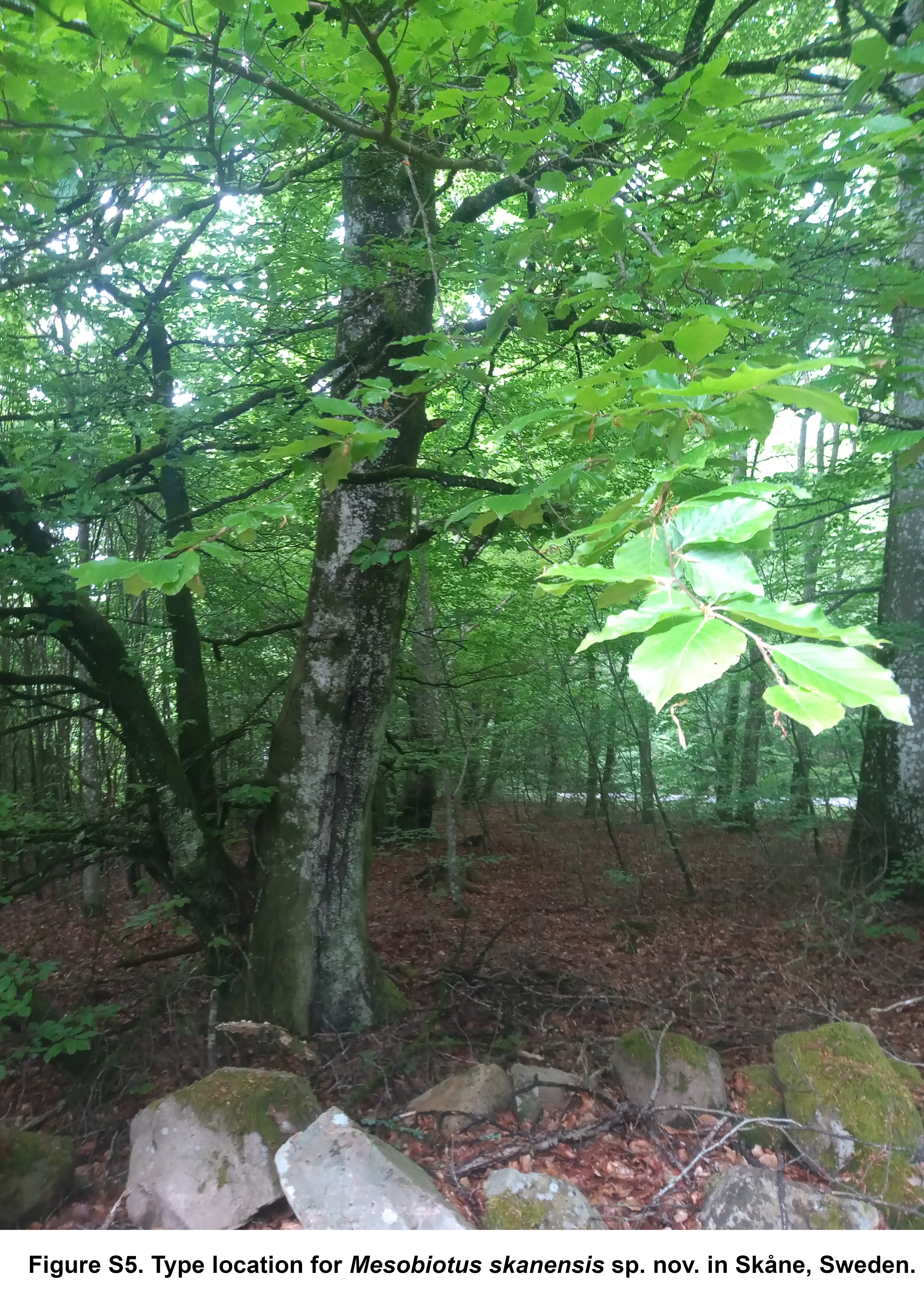

Supplement: Supplementary file 9 — Supplementary Material 9 [file 41598_2025_88063_MOESM9_ESM.jpeg]

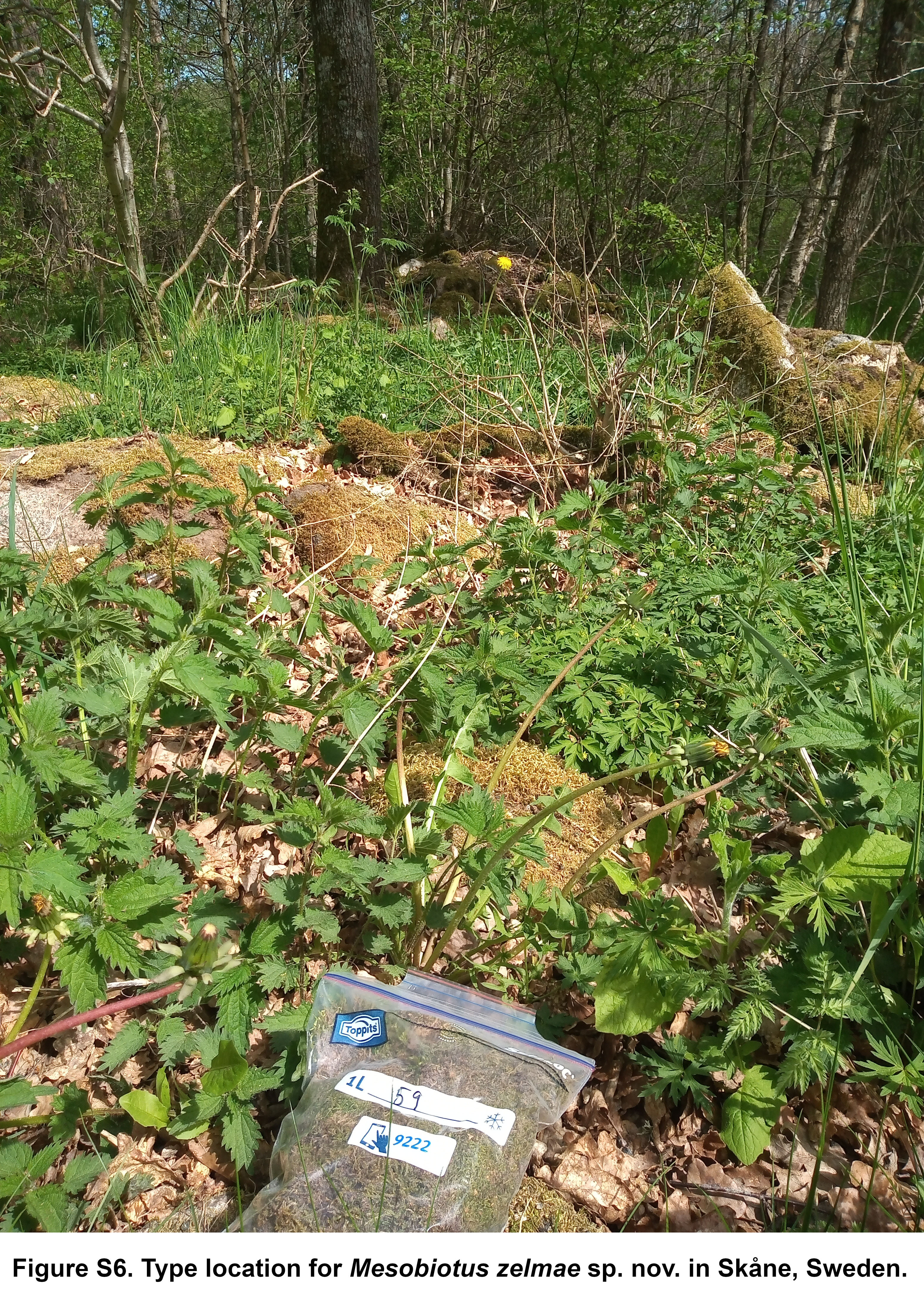

Supplement: Supplementary file 10 — Supplementary Material 10 [file 41598_2025_88063_MOESM10_ESM.jpeg]
